# Hydrogen-Bonding Motifs in Adducts of Allylamine with the 10 Simplest *n*-Alcohols: Single-Crystal X-ray Diffraction Studies and Computational Analysis

**DOI:** 10.1021/acs.cgd.2c00316

**Published:** 2022-10-11

**Authors:** Bernadeta Prus, Michał K. Cyrański, Roland Boese, Janusz Zachara, Łukasz Dobrzycki

**Affiliations:** † Laboratory of Advanced Crystal Engineering, Faculty of Chemistry, University of Warsaw, Żwirki i Wigury 101, 02-089Warsaw, Poland; ‡ Faculty of Chemistry, Warsaw University of Technology, ul. Noakowskiego 3, 00-664Warsaw, Poland

## Abstract

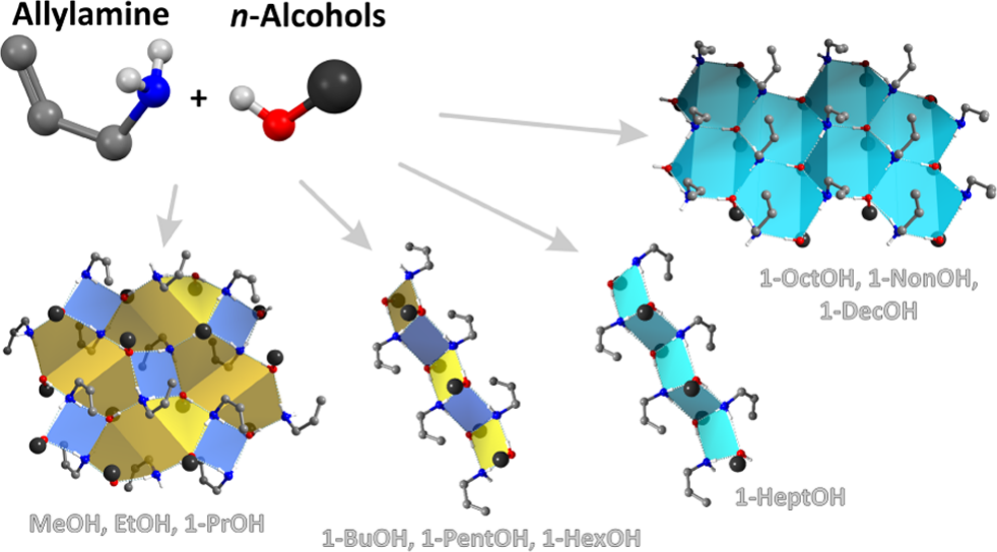

In this paper, we analyzed the homologous series of 10 allylamine adducts with *n*-alcohols from methanol to decanol. These are the first adduct structures containing aliphatic *n*-alcohols and an aliphatic amine as co-formers. While all of the ingredients are liquids under ambient conditions, the phases were synthesized with the use of the *in situ* crystallization technique assisted by IR laser-focused radiation at atmospheric pressure. The structures were characterized by single-crystal X-ray diffraction. All of the phases contain the amine and alcohol in a 1:1 ratio. The architecture of the structures, based on hydrogen-bonding interactions between NH_2_ and OH moieties, depends on the size of the alcohol and changes in a systematic way. The three smallest alcohol adducts contain centrosymmetric layers of molecules of the L4(4)8(8) type. The next four alcohol adducts have the T4(2) topology. The structures with the biggest alcohols contain non-centrosymmetric L6(6) layers. The structural investigations were supported by periodic DFT calculations at the B3LYP/pobTZVP level. The cohesive and adhesive energies made up of layer (*E*_lbe_) and ribbon (*E*_rbe_) binding energies were used to predict which type of architecture can be formed. The thermal stabilities of the adducts correlate with the melting points of the co-forming alcohols, with no evident relation to the adduct architecture.

## Abstract 2

In this paper, we analyzed the homologous series of 10 allylamine adducts with *n*-alcohols from methanol to decanol. These are the first adduct structures containing the aliphatic *n-*alcohols and the aliphatic amine as co-formers. The architecture of the crystals depends on the size of the alcohol and changes in a systematic way.

## Introduction

Crystal engineering is nowadays one of the most rapidly expanding disciplines within crystallography and solid-state sciences. Although this term was first used a long time ago,^1^ it was later popularized by Desiraju.^2^ A literature survey concerning some historical aspects of crystal engineering can be found in the paper by Braga.^3^ In general, crystal engineering is a way to rationally design crystal structures using all possible intra- and intermolecular interactions. An example of using such interactions leading to the formation of the desired molecular arrangements is crystals of differently substituted cinnamic acid derivatives.^4,5^ Of course, more crystal architectures may be obtained when different compounds are used, giving so-called cocrystals.^6−12^ To enhance the rational design of novel solid-state phases, analysis of the possible intermolecular interactions, *i.e.*, “*supramolecular synthons*”^13^ is required. Among the most important supramolecular synthons, hydrogen bonding and π–π stacking should be mentioned. Although these interactions are rather weak, they can effectively govern the structure of molecular crystals in the absence of stronger forces (covalent, ionic, etc.).

Very important and popular functional groups in organic chemistry are OH and NH_2_ moieties. Furthermore, these groups, with respect to their possible interactions, seem to be complementary.^14^ Surprisingly, a CSD [14] search of OH···NH_2_ synthons with the following criteria gave less than 2000 structures: N···O distance in 2.5–3.5 Å range, NH_2_ amidic and OH carboxylic interactions excluded, only nonmetals, −NH_2_ has to be neutral, and N bonded to three atoms only. Most of these examples correspond to aromatic systems with functional groups located in the same^15−17^ or different^18−20^ molecules. There are limited numbers of examples regarding aliphatic systems, and these mostly concern diamine and diol cocrystals.^21−24^ However, some systematic research on cocrystal formation of substituted diols with different monoamines also exists.^25^ In crystals with these OH···NH_2_ contacts, different topologies of hydrogen bonds (HBs) can be found, such as finite threecenter interactions,^26,27^ one-dimensional chains or ribbons,^24,28,29^ and molecules arranged in hydrogen-bonded layers.^30^ In many cases, the crystals contain relatively large molecules, often with additional substituents that can affect the whole structure. On the other hand, simple aliphatic or even aromatic compounds containing single NH_2_ or OH groups are liquid under ambient conditions. This can make the crystallization experiments and further structural analysis complicated. Yet, there are a few papers dedicated to ambient pressure structural investigations of such compounds like aliphatic aminols^31^ or cocrystallization of cresols with aniline and fluoroanilines.^32^ Increasing the pressure may also lead to successful crystallization of liquids like 3-aminopropanol.^33^ The formation of a cocrystal has to be an energetically favored process, of course. Hence, the use of various theoretical methods can be helpful, especially in the analysis of hydrogen-bonding motifs and synthon energies in the crystalline phases based on various approaches.^34−45^ However, the thermal stability of a resulting multicomponent phase does not necessarily need to be greater than the pure components. Indeed, a recent analysis of pharmaceutical cocrystals based on literature searching showed that more than 85% of such cocrystal systems have melting points between or lower than their corresponding pure mere components.^46^

In our work, we decided to focus on the cocrystallization of the simplest stable and unsaturated amine, *i.e.*, allylamine with aliphatic, primary *n-*alcohols containing 1 to 10 carbon atoms in the chain. Furthermore, the experiments were supported by periodic DFT calculations to estimate energies between interacting moieties. In such model systems, both intra- and intermolecular interactions are not affected by the presence of additional substituents and functional groups. The low molecular weight and aliphatic character of the investigated amine–alcohol binary systems indicate that these mixtures are liquid under ambient conditions, which is the main reason such simple-compound multicomponent crystals are hardly represented in the CSD. Moreover, no examples of aliphatic monoamine–monoalcohol systems are even known. In the following chapters, all of the obtained multicomponent allylamine–alcohol crystalline phases will be described as adducts.

## Experimental Section

### Crystallization and Single-Crystal X-ray Diffraction Experiments

The amine mixtures with methanol, ethanol, 1-propanol, 1-butanol, 1-pentanol, 1-hexanol, and 1-heptanol were prepared in a 1:1 molar ratio. From the analogous solution containing 1-octanol, pure alcohol crystallized with no formation of the wanted adducts. For this reason, for 1octanol, 1-nonanol, and 1-decanol, the molar ratio was changed to 2:1. All of these mixtures were liquids at room temperature and were thus sealed in thin wall glass capillaries and solidified at a lower temperature and under ambient pressure directly on a goniometer of a Bruker D8 Venture single-crystal diffractometer. Samples suitable for the diffraction experiments were obtained using the IR laser-assisted zone melting *in situ* crystallization technique.^47^ Data were collected using a Mo sealed tube and a TRIUMPH monochromator with a Photon II detector. Measurements were performed using the φ scan method with the capillary oriented parallel to the vertically mounted LT device to avoid sample destruction during the measurement. For this reason, the final data completeness was lower than the recommended 98% for some datasets giving alerts A or B in the checkCIF reports for the adducts with MeOH, EtOH, 1-PrOH, 1-BuOH, 1-HexOH, and 1-HeptOH. In almost all of the structures, except for the system containing 1-BuOH, the crystal density is lower than 1 g·cm^–3^, which causes additional alert B in the report. Each of the obtained crystals was measured at two temperatures—first, the same as during the *in situ* crystallization, and later, at 100 K or 130 K. All data were processed using the Bruker suite of programs^48−50^ and the structures were solved by direct methods and refined with the SHELX program suite.^51,52^ Because, in all of the cases, the obtained phases were oligocrystalline during the data integration, the box sizes were not refined. All O, N, and C atoms were refined anisotropically. All amine and hydroxyl group hydrogen atoms were refined without constraints, together with their isotropic displacement parameters. All C–H hydrogen atoms were placed in calculated positions and refined within the riding model. The isotropic displacement parameters were set 1.2 and 1.5 times bigger than the corresponding heavy atom for sp^2^ and sp^3^ H atoms, respectively. The atomic scattering factors were taken from the International Tables.^53^ Diamond 4^54^ and Mercury 2020.2.0 software^55^ were used for preparing the figures. The crystal structures were deposited at the Cambridge Crystallographic Data Centre with the following numbers: CCDC 2090851–2090870. These data can be obtained free of charge from www.ccdc.cam.ac.uk/structures.

### Periodic Calculations

All calculations corresponding to the obtained crystal structures were performed at the DFT(B3LYP/pob-TZVP) level of theory^56,57^ in the CRYSTAL program (CRYSTAL17 version).^58,59^ Geometry optimization was carried out for the structures determined at a lower temperature (100 or 130 K depending on the adducts) with fixed unit cell parameters. The atomic positions were allowed to vary. For the optimized crystal structures, cohesive and adhesion energies, as well as other interaction energies, were calculated. The results were corrected for dispersion (D3 dispersion energy)^60,61^ and basis set superposition error (BSSE).^62^ For molecules isolated from the crystals/layers/ribbons, ghost atoms for BSSE estimation were selected with the distance restriction—5 Å at most. For layer–layer interactions, the adjacent layer was treated as ghosts. Ribbon–ribbon interactions were not calculated, but their values were approximated using the dependencies proven for layers.^63^ Cohesive energies, interlayer interaction energies, and layer binding energies were computed according to the approach presented previously. Additionally, the **r**ibbon **b**inding **e**nergy—*E*_rbe_ (energy difference between the ribbon and corresponding molecules in the gas phase)—was introduced analogously to the layer binding energy in the following form
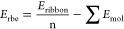


**I**nter**l**ayer **i**nteraction **e**nergy (*E*_ilie_) may be also applied in the case of ribbon (inter-ribbon **i**nteraction **e**nergy—*E*_irie_) according to the formula
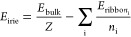
where *n* and *Z* are the numbers of molecular units in the rod and crystal unit cells, respectively. Energies of the ribbons were obtained for the same geometry as in an optimized crystal.

For referring to the contribution of each interaction to the *E*_coh_, calculations of energies per single amine–alcohol molecular pair were performed. The energies of HBs (denoted as *E*_pair_) were calculated as the difference between the energy of the dimer and the energies of the isolated molecules, according to the convention that the bonding energy is negative. For all pairs of molecules occurring in the studied adducts, structural motifs (ribbons and layers) not present in the structures were created using the edit structure function in the Mercury program.^55^ These systems were optimized allowing full relaxation, including unit cell parameters, and restricted to the original layer or rod group of symmetry. To be able to compare these created one-dimensional (1D) or two-dimensional (2D) structural motifs with those observed in the adducts, additional optimization of ribbons and layers was required in the latter case.

### Molecular Geometry *Ab Initio* Calculations

The single-amine molecule geometry optimization was performed in Gaussian09^64^ together with single-point calculations with 2π rotation of amine and vinyl groups in 2° steps preserving the C_1_ point group symmetry and without further optimization. During these calculations, the DFT method^56,57^ and B3LYP functional were used with the def2-TZVP basis set.

## Results and Discussion

### Single-Crystal X-ray Ddiffraction

**Figure 1:**
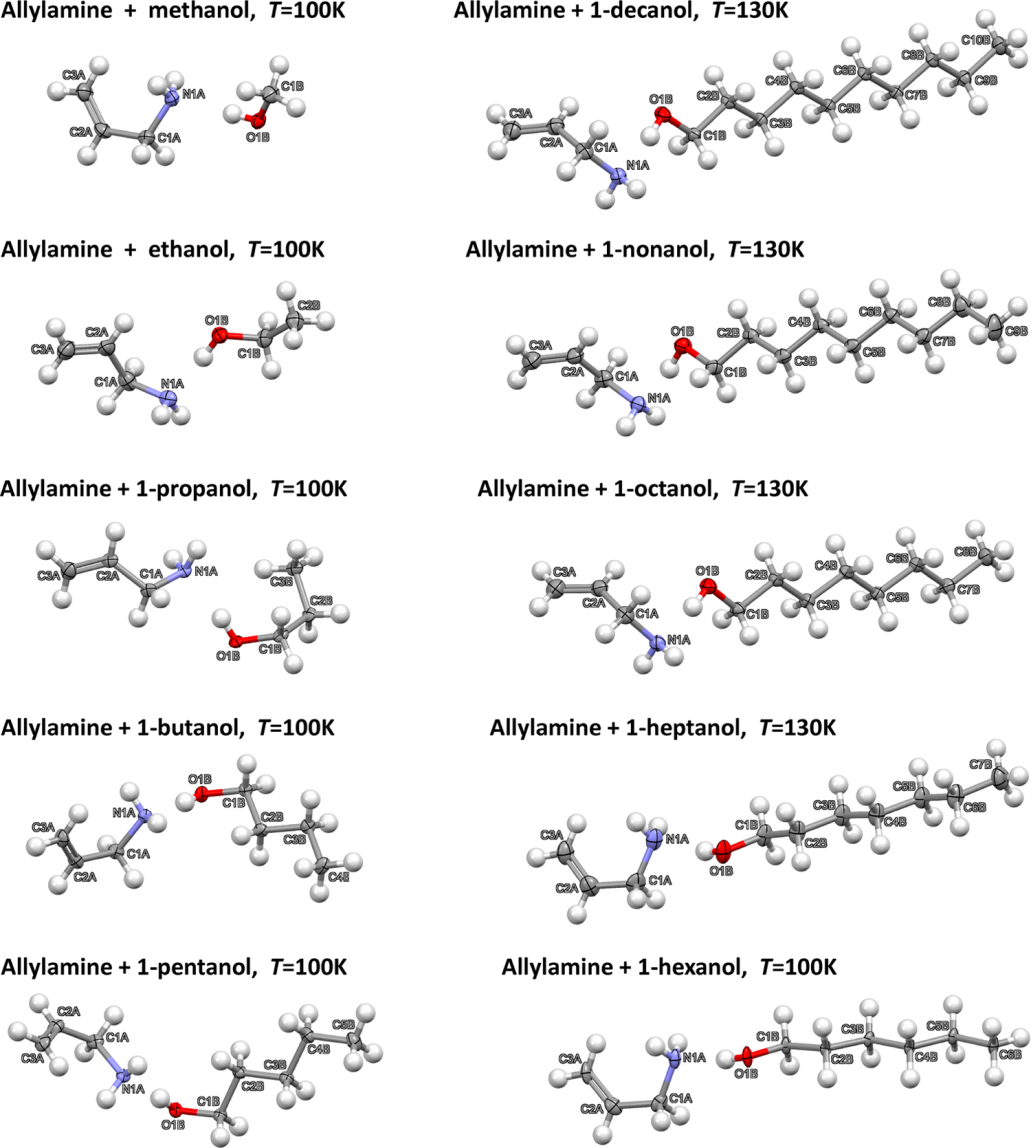
Displacement ellipsoid plots (drawn at 50% probability level) and numbering scheme for the allylamine *n*-alcohol adducts measured at either 100 or 130 K.

**Figure 2:**
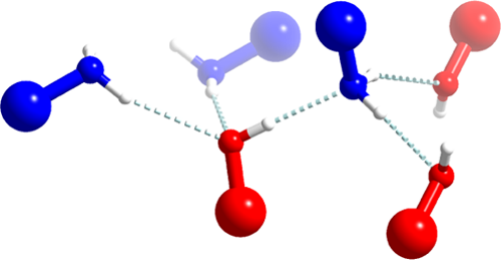
Hydrogen bond formation in the allylamine *n*-alcohol adducts.

Ten adducts of allylamine and *n*-alcohols ranging from methanol to decanol were obtained. The crystal data and the refinement parameters are presented in Table 1 together with the measurement temperatures. In the given temperature ranges, no phase transitions were observed. In the case of the cocrystal with octanol, where both low- and high-temperature data differ by only 30 K, additional measurement at 190 K also indicated no phase transition. Due to their lower quality, these data are not included in the table. All of the obtained structures are ordered at the given temperature, including the hydrogen atoms of amino and hydroxyl groups. However, at higher temperatures, the thermal displacement ellipsoids of heavy atoms indicate some libration. This is especially visible for the vinyl group in the adducts with ethanol and 1-hexanol measured at higher temperatures. In all of the structures, the asymmetric part of the unit cell contains a single amine–alcohol molecular pair, as shown in Figure 1. Atomic displacement parameters at higher temperatures are presented in Figure 1S in the ESI. In all of the structures obtained, each molecule forms three hydrogen bonds as schematically shown in Figure 2. The shortest HBs occur, as expected, between O···N atoms with the hydroxyl group acting as the donor. The remaining HBs with oxygen as the acceptor are significantly longer (see Table 1S in the ESI). These values correspond well to calculated HB energies collected in the last column of Table 1S in the ESI. The bonds of OH···N type are approximately twice as strong (energetically) as the NH···O ones. Depending on the alcohol size, the structural architecture of the crystal changes in a stepwise manner.

**Table 1:** Crystal Data and Refinement Parameter for the Obtained Structures

adduct	A + MeOH 100 K	A + MeOH 170 K	A + EtOH 100 K	A + EtOH 170 K	A + 1-PrOH 100 K	A + 1-PrOH 170 K	A + 1-BuOH 100 K	A + 1-BuOH 175 K	A + 1-PentOH 100 K	A + 1-PentOH 180 K
										
formula	C_4_H_11_NO	C_4_H_11_NO	C_5_H_13_NO	C_5_H_13_NO	C_6_H_15_NO	C_6_H_15_NO	C_7_H_17_NO	C_7_H_17_NO	C_8_H_19_NO	C_8_H_19_NO
*M*_*x*_ (g·mol^–1^)	89.14	89.14	103.16	103.16	117.19	117.19	131.21	131.21	145.24	145.24
*T* (K)	100(2)	170(2)	100(2)	170(2)	100(2)	160(2)	100(2)	175(2)	100(2)	180(2)
λ (Å)	0.71073	0.71073	0.71073	0.71073	0.71073	0.71073	0.71073	0.71073	0.71073	0.71073
crystal size (mm)	0.40 × 0.40 × 0.60	0.40 × 0.40 × 0.60	0.40 × 0.40 × 0.60	0.40 × 0.40 × 0.60	0.40 × 0.40 × 0.60	0.40 × 0.40 × 0.60	0.40 × 0.40 × 0.60	0.40 × 0.40 × 0.60	0.40 × 0.40 × 0.60	0.40 × 0.40 × 0.60
space group	**P**2_1_/*c*	**P**2_1_/*c*	**P**2_1_/*c*	**P**2_1_/*c*	**P**2_1_/*c*	**P**2_1_/*c*	**P**2_1_/*c*	**P**2_1_/*c*	**P**2_1_/*c*	**P**2_1_/*c*
unit cell dimensions										
*a* (Å)	8.8649(6)	8.9245(7)	10.6217(7)	10.6370(5)	10.1786(4)	10.2531(4)	7.4043(4)	7.4924(5)	5.0715(2)	5.0913(2)
*b* (Å)	8.0966(5)	8.1809(6)	7.7790(4)	7.9812(3)	8.7383(3)	8.7707(3)	5.1909(2)	5.1985(2)	7.4834(5)	7.5615(4)
*c* (Å)	9.0864(6)	9.1385(7)	9.3637(6)	9.2850(4)	9.2692(3)	9.3164(3)	22.2504(12)	22.3147(14)	25.7703(16)	25.7451(14)
β (deg)	113.715(2)	113.949(2)	115.521(2)	114.9541(13)	109.2120(10)	109.4430(10)	93.065(2)	93.218(2)	94.247(2)	93.8210(10)
*V* (Å^3^, *Z*)	597.11(7), 4	609.76(8), 4	698.20(7), 4	714.67(5), 4	778.52(5), 4	790.02(5), 4	853.97(7), 4	867.77(9), 4	975.35(10), 4	988.93(8), 4
*D*_*x*_ (g·cm^–3^)	0.992	0.971	0.981	0.959	1.000	0.985	1.021	1.004	0.989	0.976
*μ* (mm^–1^)	0.07	0.069	0.067	0.066	0.067	0.066	0.067	0.066	0.064	0.063
*F*(000)	200	200	232	232	264	264	296	296	328	328
θ_min_, θ_max_ (deg)	3.51, 25.05	3.49, 25.04	3.37, 25.04	3.31, 25.04	3.15, 25.05	3.14, 25.05	3.23, 25.05	3.19, 25.05	2.83, 25.05	2.81, 25.04
index range	–10 ≤ *h* ≤ 10	–10 ≤ *h* ≤ 10	–12 ≤ *h* ≤ 12	–12 ≤ *h* ≤ 12	–12 ≤ *h* ≤ 12	–12 ≤ *h* ≤ 12	–8 ≤ *h* ≤ 8	–6 ≤ *h* ≤ 8	–5 ≤ *h* ≤ 5	–5 ≤ *h* ≤ 5
	–9 ≤ *k* ≤ 8	–9 ≤ *k* ≤ 9	–9 ≤ *k* ≤ 9	–9 ≤ *k* ≤ 9	–10 ≤ *k* ≤ 10	–10 ≤ *k* ≤ 10	–5 ≤ *k* ≤ 5	–5 ≤ *k* ≤ 5	–8 ≤ *k* ≤ 8	–9 ≤ *k* ≤ 9
	–10 ≤ *l* ≤ 10	–10 ≤ *l* ≤ 10	–11 ≤ *l* ≤ 11	–11 ≤ *l* ≤ 10	–10 ≤ *l* ≤ 10	–10 ≤ *l* ≤ 10	–26 ≤ *l* ≤ 26	–26 ≤ *l* ≤ 26	–30 ≤ *l* ≤ 30	–30 ≤ *l* ≤ 30
reflections collected/independent	6276/1009	4665/1036	7149/1226	7907/1242	8080/1301	8207/1324	8616/1451	5615/1442	9714/1663	10070/1691
	*R*_*int*_ = 0.0135	*R*_*int*_ = 0.0154	*R*_*int*_ = 0.0636	*R*_*int*_ = 0.0686	*R*_*int*_ = 0.0528	*R*_*int*_ = 0.0443	*R*_*int*_ = 0.0473	*R*_*int*_ = 0.0423	*R*_*int*_ = 0.0493	*R*_*int*_ = 0.0458
completeness	95.10%	95.20%	98.60%	98.40%	94.70%	94.40%	95.80%	93.90%	97.40%	97.40%
absorption correction	multiscan	multiscan	multiscan	multiscan	multiscan	multiscan	multiscan	multiscan	multiscan	multiscan
*T*_max_, *T*_min_	0.972, 0.959	0.973, 0.960	0.974, 0.961	0.974, 0.962	0.974, 0.961	0.974, 0.961	0.974, 0.961	0.974, 0.961	0.975, 0.963	0.975, 0.963
structure solution	direct methods	direct methods	direct methods	direct methods	direct methods	direct methods	direct methods	direct methods	direct methods	direct methods
refinement method	full-matrix	full-matrix	full-matrix	full-matrix	full-matrix	full-matrix	full-matrix	full-matrix	full-matrix	full-matrix
	LSQ on *F*^2^	LSQ on *F*^2^	LSQ on *F*^2^	LSQ on *F*^2^	LSQ on *F*^2^	LSQ on *F*^2^	LSQ on *F*^2^	LSQ on *F*^2^	LSQ on *F*^2^	LSQ on *F*^2^
data/restraints/parameters	1009/0/68	1036/0/68	1226/0/77	1242/0/78	1301/0/87	1324/0/87	1451/0/96	1442/0/96	1663/0/105	1691/0/105
GOF on *F*^2^	1.054	1.061	1.063	1.143	1.062	1.056	1.051	1.056	1.087	1.076
final *R* indices	949 data; *I* > 2*σ*(*I*)	938 data; *I* > 2*σ*(*I*)	1040 data; *I* > 2*σ*(*I*)	1042 data; *I* > 2*σ*(*I*)	1208 data; *I* > 2*σ*(*I*)	1225 data; *I* > 2*σ*(*I*)	1313 data; *I* > 2*σ*(*I*)	1250 data; *I* > 2*σ*(*I*)	1510 data; *I* > 2*σ*(*I*)	1498 data; *I* > 2*σ*(*I*)
	*R*1 = 0.0314	*R*1 = 0.0367	*R*1 = 0.0473	*R*1 = 0.0579	*R*1 = 0.0355	*R*1 = 0.0343	*R*1 = 0.0364	*R*1 = 0.0390	*R*1 = 0.0384	*R*1 = 0.0418
	*w*R**2 = 0.0836	*w*R**2 = 0.1083	*w*R**2 = 0.1192	*w*R**2 = 0.1526	*w*R**2 = 0.0941	*w*R**2 = 0.0848	*w*R**2 = 0.0909	*w*R**2 = 0.0983	*w*R**2 = 0.0980	*w*R**2 = 0.1119
	all data	all data	all data	all data	all data	all data	all data	all data	all data	all data
	*R*1 = 0.0333	*R*1 = 0.0397	*R*1 = 0.0569	*R*1 = 0.0653	*R*1 = 0.0381	*R*1 = 0.0371	*R*1 = 0.0407	*R*1 = 0.0468	*R*1 = 0.0421	*R*1 = 0.0466
	*w*R**2 = 0.0862	*w*R**2 = 0.1126	*w*R**2 = 0.1297	*w*R**2 = 0.1632	*w*R**2 = 0.0973	*w*R**2 = 0.0882	*w*R**2 = 0.0966	*w*R**2 = 0.1067	*w*R**2 = 0.1019	*w*R**2 = 0.1182
extinction coefficient				0.18(4)	0.021(6)	0.032(6)	0.035(6)	0.072(8)	0.034(7)	0.056(10)
ρ_max_, ρ_min_ (eÅ^–3^)	0.178, −0.147	0.169, −0.139	0.210, −0.185	0.231, −0.254	0.218, −0.193	0.187, −0.135	0.211, −0.170	0.167, −0.136	0.264, −0.171	0.190, −0.169
										

adduct	A + 1-HexOH 100 K	A + 1-HexOH 190 K	A + 1-HeptOH 130 K	A + 1-HeptOH 185 K	A + 1-OctOH 130 K	A + 1-OctOH 160 K	A + 1-NonOH 130 K	A + 1-NonOH 200 K	A + 1-DecOH 130 K	A + 1-DecOH 225 K
formula	C_9_H_21_NO	C_9_H_21_NO	C_10_H_23_NO	C_10_H_23_NO	C_11_H_25_NO	C_11_H_25_NO	C_12_H_27_NO	C_12_H_27_NO	C_13_H_29_NO	C_13_H_29_NO
*M*_*x*_ (g·mol^–1^)	159.27	159.27	173.29	173.29	187.32	187.32	201.34	201.34	215.37	215.37
*T* (K)	100(2)	190(2)	130(2)	185(2)	130(2)	160(2)	130(2)	200(2)	130(2)	225(2)
λ (Å)	0.71073	0.71073	0.71073	0.71073	0.71073	0.71073	0.71073	0.71073	0.71073	0.71073
crystal size (mm)	0.40 × 0.40 × 0.60	0.40 × 0.40 × 0.60	0.40 × 0.40 × 0.60	0.40 × 0.40 × 0.60	0.40 × 0.40 × 0.60	0.40 × 0.40 × 0.60	0.40 × 0.40 × 0.60	0.40 × 0.40 × 0.60	0.40 × 0.40 × 0.60	0.40 × 0.40 × 0.60
space group	*P*1̅	*P*1̅	**P**2_1_/**c**	**P**2_1_/**c**	**P**2_1_/**c**	**P**2_1_/**c**	**C**2/c	**C**2/**c**	**P**2_1_/*c*	**P**2_1_/*c*
unit cell dimensions										
*a* (Å)	5.2111(2)	5.2398(2)	9.9676(6)	10.0422(7)	30.1799(14)	30.2054(12)	67.013(3)	67.776(3)	34.416(3)	34.727(3)
*b* (Å)	7.8019(5)	7.9107(5)	5.3328(2)	5.3397(2)	4.9110(2)	4.9125(2)	4.9365(2)	4.9635(2)	4.9010(3)	4.9300(3)
*c* (Å)	13.1238(9)	13.2024(8)	22.1687(13)	22.2607(14)	8.5700(3)	8.5777(3)	8.3348(3)	8.3696(4)	8.5892(7)	8.6883(8)
α (deg)	91.248(2)	91.035(2)								
β (deg)	90.048(2)	89.248(2)	93.445(2)	92.8860(19)	93.986(2)	93.8000(10)	90.3320(10)	92.729(2)	96.057(3)	96.715(3)
γ (deg)	94.420(2)	93.798(2)								
*V* (Å^3^, *Z*)	531.85(5), 2	545.92(5), 2	1176.25(11), 4	1192.16(12), 4	1267.12(9), 4	1270.00(8), 4	2757.2(2), 8	2812.4(2), 8	1440.68(19), 4	1477.3(2), 4
*D*_*x*_ (g·cm^–3^)	0.995	0.969	0.979	0.966	0.982	0.980	0.970	0.951	0.993	0.968
μ/mm^–1^	0.063	0.062	0.062	0.061	0.061	0.061	0.06	0.059	0.061	0.059
*F*(000)	180	180	392	392	424	424	912	912	488	488
θ_min_, θ_max_ (deg)	3.02, 25.04	2.58, 25.05	3.68, 25.05	4.06, 25.04	3.38, 25.05	3.38, 25.04	3.04, 25.05	3.01, 25.05	2.98, 25.05	2.95, 25.04
index range	–5 ≤ *h* ≤ 5	–5 ≤ *h* ≤ 5	–11 ≤ *h* ≤ 11	–11 ≤ *h* ≤ 11	–35 ≤ *h* ≤ 35	–35 ≤ *h* ≤ 35	–78 ≤ *h* ≤ 78	–80 ≤ *h* ≤ 80	–41 ≤ *h* ≤ 41	–41 ≤ *h* ≤ 41
	–9 ≤ *k* ≤ 9	–9 ≤ *k* ≤ 9	–5 ≤ *k* ≤ 5	–5 ≤ *k* ≤ 5	–5 ≤ *k* ≤ 5	–5 ≤ *k* ≤ 5	–5 ≤ *k* ≤ 5	–5 ≤ *k* ≤ 5	–5 ≤ *k* ≤ 5	–5 ≤ *k* ≤ 5
	–15 ≤*l* ≤ 15	–15 ≤*l* ≤ 15	–26 ≤*l* ≤ 26	–26 ≤*l* ≤ 26	–10 ≤*l* ≤ 10	–10 ≤*l* ≤ 10	–9 ≤*l* ≤ 9	–9 ≤*l* ≤ 9	–10 ≤*l* ≤ 10	–10 ≤*l* ≤ 10
reflections collected/independent	5664/1767	6493/1815	12542/1972	10808/2001	11874/2223	11989/2223	12696/2418	13382/2461	13327/2512	14862/2573
	*R*_int_ = 0.0466	*R*_int_ = 0.0526	*R*_int_ = 0.0811	*R*_int_ = 0.0730	*R*_int_ = 0.0634	*R*_int_ = 0.0572	*R*_int_ = 0.0484	*R*_int_ = 0.0421	*R*_int_ = 0.0683	*R*_int_ = 0.0701
completeness	93.80%	93.90%	94.40%	94.30%	99.20%	99.00%	99.50%	99.40%	98.30%	98.50%
absorption correction	multiscan	multiscan	multiscan	multiscan	multiscan	multiscan	multiscan	multiscan	multiscan	multiscan
*T*_max_, *T*_min_	0.975, 0.963	0.976, 0.964	0.976, 0.964	0.976, 0.964	0.976, 0.964	0.976, 0.964	0.976, 0.965	0.977, 0.966	0.976, 0.964	0.977, 0.965
structure solution	direct methods	direct methods	direct methods	direct methods	direct methods	direct methods	direct methods	direct methods	direct methods	direct methods
refinement method	full-matrix	full-matrix	full-matrix	full-matrix	full-matrix	full-matrix	full-matrix	full-matrix	full-matrix	full-matrix
	LSQ on *F*^2^	LSQ on *F*^2^	LSQ on *F*^2^	LSQ on *F*^2^	LSQ on *F*^2^	LSQ on *F*^2^	LSQ on *F*^2^	LSQ on *F*^2^	LSQ on *F*^2^	LSQ on *F*^2^
data/restraints/parameters	1767/0/113	1815/0/114	1972/0/123	2001/0/122	2223/0/132	2223/0/132	2418/0/141	2461/0/141	2512/0/150	2573/0/150
GOF on *F*^2^	1.129	1.145	1.136	1.107	1.124	1.161	1.068	1.064	1.124	1.12
final *R* indices	1485 data; *I* > 2*σ*(*I*)	1483 data; *I* > 2*σ*(*I*)	1649 data; *I* > 2*σ*(*I*)	1578 data; *I* > 2*σ*(*I*)	1839 data; *I* > 2*σ*(*I*)	1798 data; *I* > 2*σ*(*I*)	2018 data; *I* > 2*σ*(*I*)	1881 data; *I* > 2*σ*(*I*)	1936 data; *I* > 2*σ*(*I*)	1705 data; *I* > 2*σ*(*I*)
	*R*1 = 0.0490	*R*1 = 0.0575	*R*1 = 0.0782	*R*1 = 0.0801	*R*1 = 0.0534	*R*1 = 0.0513	*R*1 = 0.0473	*R*1 = 0.0431	*R*1 = 0.0607	*R*1 = 0.0632
	*w*R**2 = 0.1368	*w*R**2 = 0.1618	*w*R**2 = 0.2176	*w*R**2 = 0.2478	*w*R**2 = 0.1538	*w*R**2 = 0.1598	*w*R**2 = 0.1324	*w*R**2 = 0.1289	*w*R**2 = 0.1904	*w*R**2 = 0.1863
	all data	all data	all data	all data	all data	all data	all data	all data	all data	all data
	*R*1 = 0.0566	*R*1 = 0.0670	*R*1 = 0.0863	*R*1 = 0.0912	*R*1 = 0.0622	*R*1 = 0.0635	*R*1 = 0.0555	*R*1 = 0.0561	*R*1 = 0.0752	*R*1 = 0.0902
	*w*R**2 = 0.1461	*w*R**2 = 0.1748	*w*R**2 = 0.2260	*w*R**2 = 0.2585	*w*R**2 = 0.1624	*w*R**2 = 0.1800	*w*R**2 = 0.1414	*w*R**2 = 0.1430	*w*R**2 = 0.2019	*w*R**2 = 0.2084
extinction coefficient		0.22(6)	0.11(2)		0.025(7)	0.022(7)	0.0033(9)	0.0034(12)	0.032(9)	0.018(6)
ρ_max_, ρ_min_ (eÅ^–3^)	0.210, −0.344	0.31′7, −0.228	0.284, −0.284	0.210, −0.253	0.300, −0.217	0.247, −0.212	0.221, −0.193	0.174, −0.180	0.291, −0.205	0.160, −0.182

**Figure 3:**
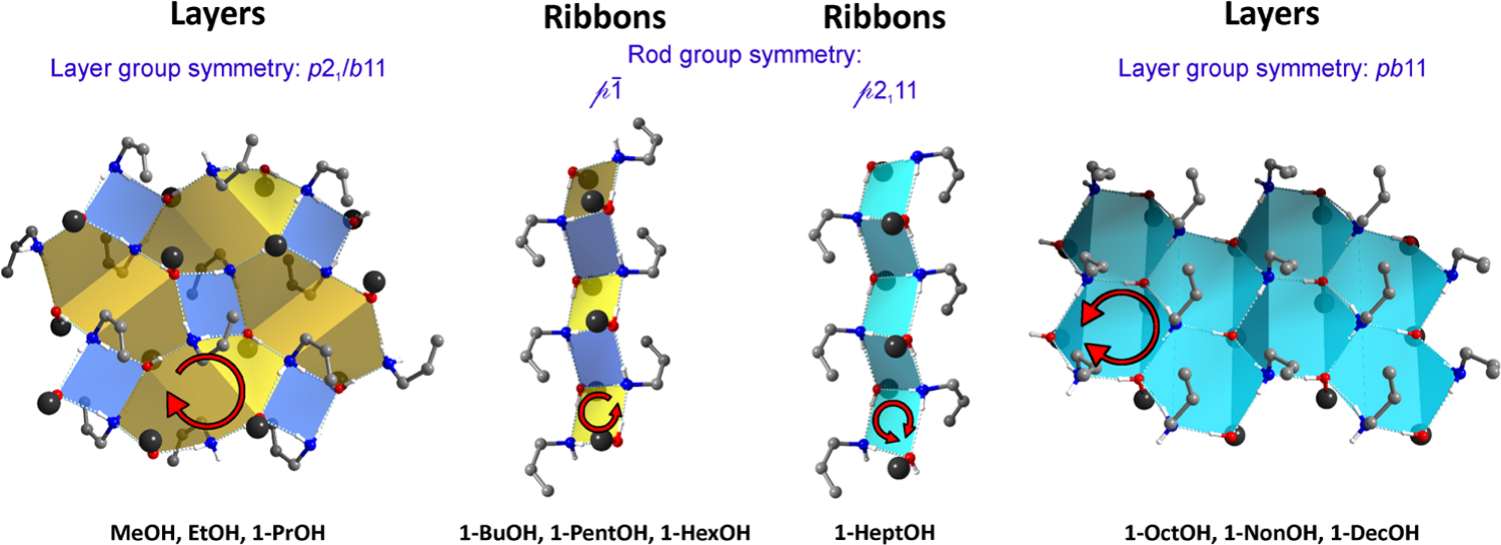
Structural motifs present in the allylamine *n*-alcohol adducts with the given layer or rod group symmetry. Carbon atom chain in alcohol molecules represented as single large black spheres.

The adducts with the first three smallest alcohols contain molecules, which are bound via the HBs in layers consisting of donors and acceptors forming four- and eight-membered rings. The next four structures (adducts with butanol, pentanol, hexanol, and heptanol) contain a stack of molecules organized in columns with N and O moieties organized in tapes of four-membered rings. The remaining three adducts have again a 2D molecular architecture with interacting amine and alcohol moieties forming sheets, where hydrogen-bonded amine and hydroxyl groups are arranged in layers built from corrugated six-membered rings. The discussed motifs are presented in Figure 3, together with either layer or rod symmetry group symbols.

**Figure 4:**
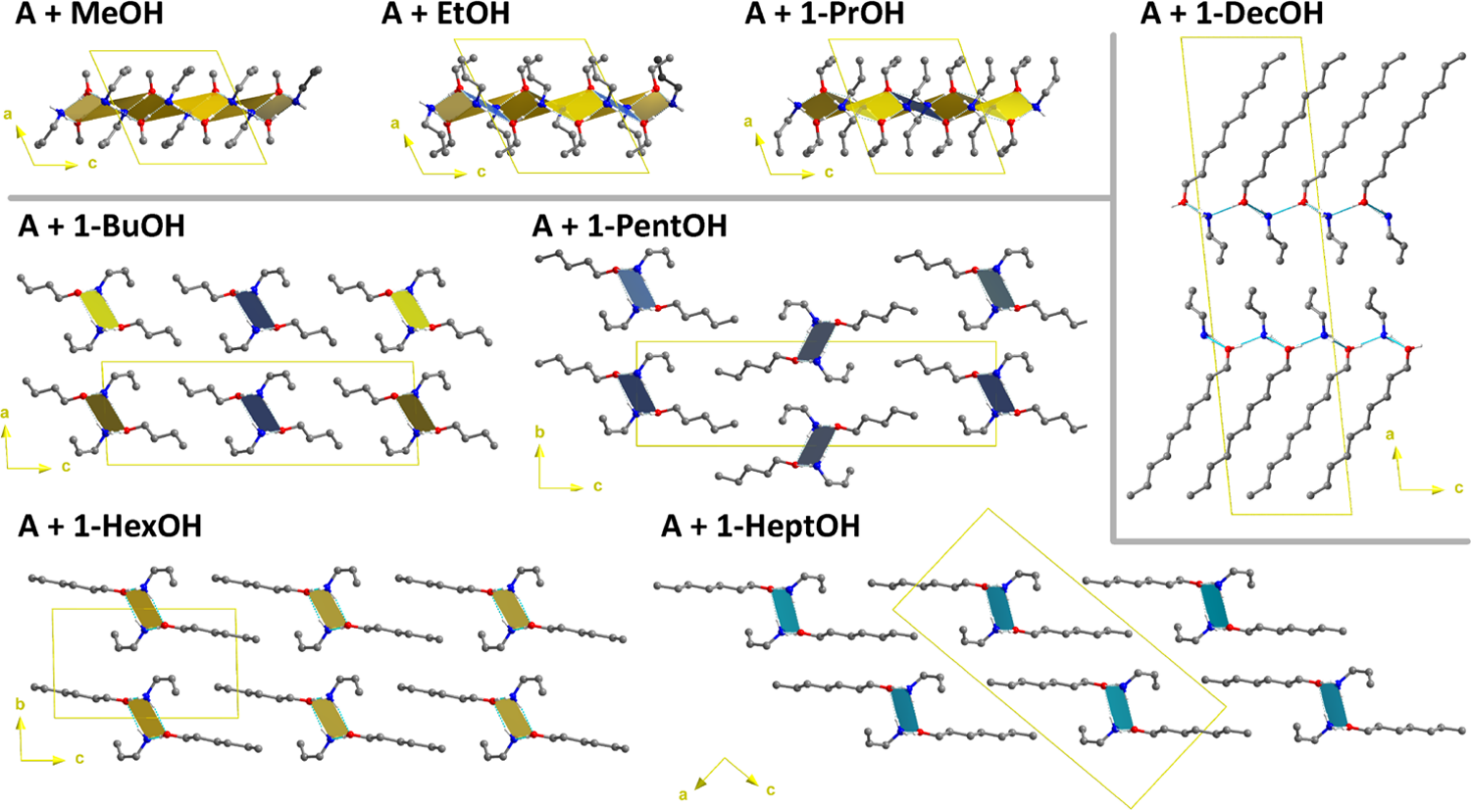
Packing diagrams of allylamine n-alcohol adducts. Aliphatic hydrogen atoms were omitted for clarity.

According to the HB motif notation proposed in reference (65), the layers in the adducts with the three smallest alcohols are denoted as L4(4)8(8), the tapes in the 1D-type structures are described as T4(2), and layers for the last three alcohols are defined as L6(6). However, such a definition of the structural motifs does not include the orientation of hydrogen atoms in the rings,^66^ which in the case of the analyzed structures is indicated by different ring colors. The yellow rings have homodromic arrangement of the H atoms—circular single-side red arrows, whereas the cyan rings have antidromic H atom orientation—circular double-side red arrows. The remaining blue rings can be considered as heterodromic: the H atoms have no particular direction. The complete description of the HB motifs in the presented structures can be based on the topological approach proposed by Etter.^67^ According to this notation, adducts containing MeOH, EtOH, and 1-PrOH have HBs with R_4_^2^(8) + R_8_^8^(16) motifs. Interestingly, in the case of the 1D HB topology, this notation differentiates ribbons present in the structures containing 1-BuOH, 1-PentOH, and 1-HexOH [R_4_^2^(8) + R_4_^4^(8)] with the adducts with 1-HeptOH, which has R_4_^3^(8) HB rings. The layered structures with the biggest alcohols have R_6_^5^(12) motifs. The packing diagrams with highlighted HB motifs are presented in Figure 4. Due to the high similarities of the 1OctOH, 1NonOH, and 1DecOH structures, only the latter structure is presented in the figure. As can be seen, the layers with R_4_^2^(8) + R_8_^8^(16) ring motifs are reserved for the smallest alcohols. This layer topology observed in some hydrates is reported here as the first example of an alcohol–amine adduct. In such a case, the molecular sheets are centrosymmetric, so both the amine and alcohol molecules are present on each side of the layer. The layer thickness is the smallest for the adducts with MeOH and, surprisingly, there is almost no difference in this parameter between ethanol- and propanol-containing crystals.

Next, in the series, 1-butanol adopts a different topology with molecules organized in 1D columns. Such a molecular packing, contrary to the 2D arrangement, can be attributed to slightly too long aliphatic chains causing large steric effects within hypothetical layers if they were formed. This alcohol, similar to the next three in the sequence, gives structures with parallel rows of molecules packed in the unit cell. Longer aliphatic chains of the alcohol cause another change in the crystal architecture, where dispersive forces between CH_2_ moieties may play a significant role. In these structures, the layers are noncentrosymmetric and all amine molecules are located on the opposite side of the alcohol moieties. The comparison of all structures with the 2D topology shows that the efficiency of the packing of the amine–alcohol molecular pairs in the layers is better in the case of the three smallest alcohols, *i.e.*, for the adducts with HBs of R_4_^2^(8) + R_8_^8^(16) motifs. The packing in layers can be described as a 2D density of molecules similar to the ρ_2D_ parameter introduced for the ionic structures.^68,69^ Indeed, the ρ_2D_ values are 0.0544, 0.549, and 0.0494 Å^–2^ for MeOH, EtOH, and 1-PrOH, respectively. In the case of the largest three alcohols [R_6_^5^(12) motifs], this parameter falls within the range 0.0475–0.0486 Å^–2^. In the structures with 1OctOH, 1NonOH, and 1-DecOH, all of the alcohols are more poorly packed in the layers than in the crystals containing corresponding hydrocarbons^70,71^ (ρ_2D_ equal to 0.0535, 0.0539, and 0.0526 Å^–2^ for 1-octane, 1-nonane, and 1-decane, respectively). Such a slightly loose molecular packing for these three adducts is balanced by the aliphatic chain tilt according to the layer. The tilt angle reaches approximately 50°. Interestingly, in these phases, the alcohol molecules are better packed in layers than for the single-component 1-octanol structure,^72^ where the ρ_2D_ parameter is 0.0458 Å^–2^ only, with the tilt angle reaching 53°.

### Periodic Calculations for the Crystal Structures

To better understand the formation of layers and columns in the obtained structures, a series of 1D–3D periodic calculations for structural motifs were performed. In the adducts with the three smallest alcohols, adjacent layers interact via weak dispersion forces. Here, the contribution of this type of interaction, denoted as interlayer interaction energy (*E*_ilie_), to the cohesive energy of the crystal is quite low, reaching about 9–10% (see Table 2). The remaining ca. 90% comes from the layer binding energy (*E*_lbe_), and this value corresponds to the thickness of the layer. Hence, for EtOH and 1-PrOH, the energy is comparable. For the columnar structures, i.e., those containing 1D HB motifs, the dispersion interactions play more important roles as *E*_irie_ is around 30% of the cohesive energy. By comparing structures in Figure 4, it can be seen that the aliphatic chains in the structures with 1-BuOH and 1-PentOH are differently oriented than in the adducts with 1-HexOH and 1-HeptOH. Based on the DFT calculations, the largest dispersive interactions occur in the latter two structures with significantly more negative *E*_irie_ values in comparison to the former two phases (see Table 2). The ribbon binding energy (*E*_rbe_) increases proportionally to the number of carbon atoms in the alcohol molecule for centrosymmetric columns, while for the adducts with 1-HeptOH containing a helical R_4_^3^(8) motif, the *E*_rbe_ is slightly higher than that for the structure with 1-HexOH. Structural motifs with layers composed of hexagonal rings are described in the literature as most favorable;^67^ however, in the series of adducts presented here with the 10 simplest *n*-alcohols, it only occurs for the three longest molecules. The interlayer interaction energies (*E*_ilie_) for the structures with hexagonal R_6_^5^(12) ring motifs are almost equal for the adducts with even numbers of carbon atoms, reaching approximately −17 kJ/mol, whereas for 1-NonOH, the *E*_ilie_ value of −10.32 kJ/mol is significantly less negative. The layer binding energy (*E*_lbe_), on the other hand, decreases significantly with the length of the chain similar to the contribution of the *E*_ilie_ to the cohesive energy.

**Table 2:** Cohesive Energies (*E*_coh_) for the Allylamine Adducts with *n*-Alcohols Together with the Contribution of the Layer/Ribbon Binding Energies (*E*_lbe_/*E*_rbe_) and Interlayer/Inter-ribbon Binding Energies (*E*_ilie_/*E*_irie_)^a^

alcohol	*E*_coh_	*E*_lbe_/E_rbe_	*E*_ilie_/*E*_irie_
MeOH	–127.35 (100%)	–112.78 (89.04%)	–13.88 (10.96%)
EtOH	–138.47 (100%)	–125.55 (90.68%)	–12.91 (9.32%)
1-PrOH	–144.48 (100%)	–128.84 (89.38%)	–15.31 (10.62%)
1-BuOH	–151.12 (100%)	–106.82 (70.69%)	–44.30 (29.31%)
1-PentOH	–155.65 (100%)	–110.84 (71.21%)	–44.82 (28.79%)
1-HexOH	–166.15 (100%)	–113.69 (68.42%)	–52.46 (31.58%)
1-HeptOH	–168.49 (100%)	–113.13 (67.14%)	–55.36 (32.86%)
1-OctOH	–181.64 (100%)	–164.28 (90.56%)	–17.12 (9.44%)
1-NonOH	–186.62 (100%)	–176.10 (94.46%)	–10.32 (5.54%)
1-DecOH	–198.10 (100%)	–180.53 (91.23%)	–17.36 (8.77%)

a Energy values given in kJ·mol^–1^.

### Periodic Calculations for the Theoretically Generated 1D and 2D Structural Motifs

**Figure 5:**
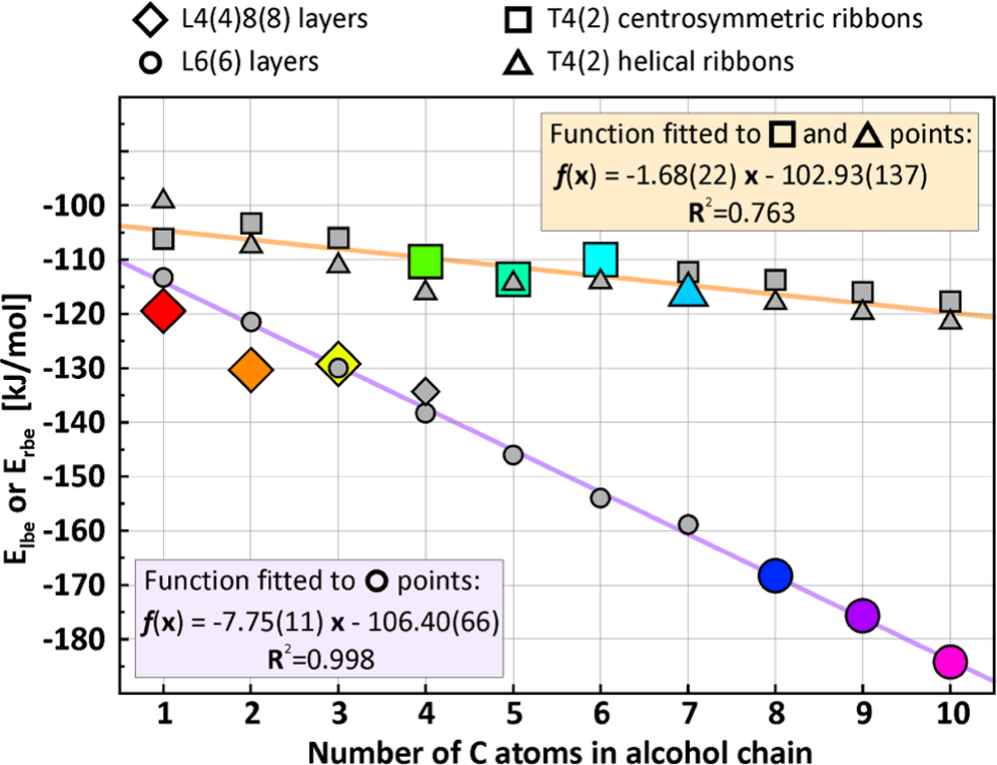
Energy calculated for observed and generated 1D and 2D structural motifs (normalized to alcohol–amine pairs), based on the allylamine–alcohol adducts. Gray points represent energies for the generated motifs, while colored ones correspond to the geometry-optimized motifs observed in the real structures.

The performed periodic calculations for the obtained structures could not explain why a particular motif is preferred for a given system and why the motifs change with an increase of the aliphatic chain length of the alcohol. To compare binding energies in layers or ribbons, every feasible structural motif was generated and optimized in the CRYSTAL17 program. Because of the steric effects in the centrosymmetric L4(4)8(8) motifs, it was possible to generate such layers for the allylamine-1-BuOH system only, whereas L6(6) layers and both types of T4(2) ribbons were reconstructed for all alcohols. Such obtained values were compared with appropriate energies for 1D or 2D motifs isolated from real structures and optimized with the preserved layer or rod symmetry groups. All of the *E*_lbe_/*E*_rbe_ values for the predicted and observed motifs are presented in Figure 5 with the corresponding values given in Table 2S in the ESI. These energies of course differ from those collected in Table 2 as the 1D or 2D motifs were retrieved from the crystals and afterward solely optimized. The points representing each motif are marked as diamonds, squares, triangles, and circles for the L4(4)8(8) layers, centrosymmetric T4(2) ribbons, helical T4(2) ribbons, and L6(6) layers, respectively. Additionally, the points corresponding to motifs observed in the real structures are color-coded: for example, red represents the adducts with methanol. The dependencies show that *E*_lbe_/*E*_rbe_ does not determine the packing directly, but its analysis may help to predict the molecular architecture. For the structures with the three smallest alcohols, the most energetically favorable L4(4)8(8) motif can be observed (for 1-PrOH, the difference is at the level of the calculation error). However, the **E**_**lbe**_ energy (Figure 5) for the adduct with 1-PrOH is comparable or even higher than for the system containing EtOH, which results from slightly too long alcohol, and may indicate the change of the architecture type for the next alcohol in the series. Indeed, the following four alcohols cocrystallize with allylamine giving adducts of 1D architecture type. The calculations do not explain the type of ribbons in the structures. In almost all cases, ribbons with 2_1_ symmetry are energetically preferred, but in reality, it occurs in the adducts with 1-heptanol only. Here, for the 1D systems, the interaction between molecules arranged in columns is critical, as the dispersive interaction between aliphatic chains constitutes about 30% of the cohesive energy in the studied adducts, whereas for the layered structures, it does not exceed 11% (Table 2).

The formation of 1D motifs for the smallest alcohols is therefore energetically unfavorable due to the expected small inter-ribbon binding energy contribution to the cohesive energy. Layers with hexagonal synthons tend to be more favorable with the elongation of the aliphatic chain; moreover, the changes are linear. Such a linear dependence of the *E*_lbe_ energy is due to the layer architecture, where all of the alcohol species are located on the same side. The function fitted to all of the points representing L6(6) motifs, both from experiment and calculations, shows that −106.40(66) kJ/mol corresponds to hydrogen bonding of hydroxyl and amine groups and amine–amine interactions, whereas the gain in layer stabilization corresponding to aliphatic chain elongation yields 7.75(11) kJ/mol per CH_**3**_ unit, so the L6(6) layers occurs in real structures for longer alcohol molecules only. The linear function can also be fitted to square and triangle points representing calculated *E*_rbe_ energies for both the observed and generated 1D motifs, including centrosymmetric and helical T4(2) ribbons. Here, the *R*^2^ parameter is much worse, which is mainly attributed to doubling the energy values for each structure. Nevertheless, the y-intercept of the curve yielding −102.93(137) kJ/mol representing amine and hydroxyl group interactions within the ribbon unit is comparable to the value obtained for L6(6) motifs. However, the slope of the curve which is equal to −1.68(22) is much smaller, meaning the increase of the chain length is not as energetically profitable as in the L6(6) layers due to interactions between alcohol/amine molecules possible in one dimension only.

### Potential Energy Surface and the Allylamine Geometry in the Adducts with *n*-Alcohols

**Figure 6:**
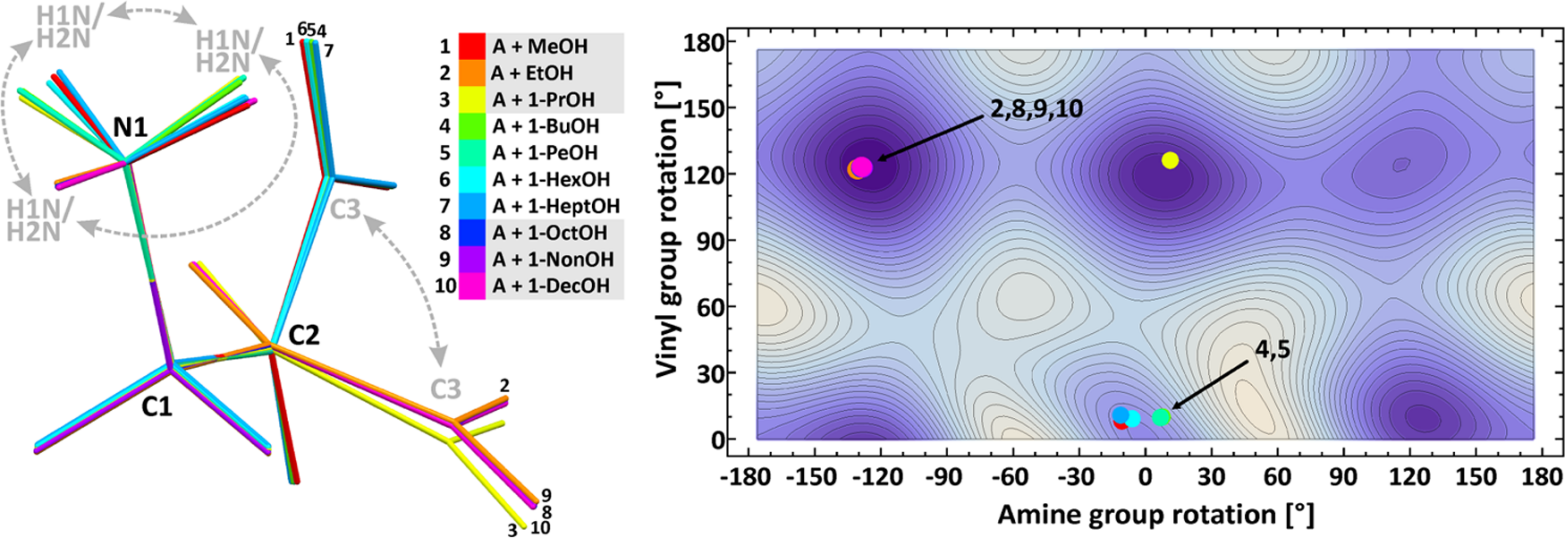
Overlay of the allylamine molecules from all of the adducts (left), potential energy surface for vinyl and amine group rotations presented as a density plot (right); the darker color corresponds to deeper minima, with contours every 1.0502 kJ/mol.

In all of the adducts, the common molecule is allylamine. The geometry of this molecule can be easily described by two dihedral angles defining the vinyl group and the amine group rotations. In the former case, this is the N1–C1–C2–C3 torsion angle (the label suffixes are omitted), whereas the latter angle is based on the centroid of the hydrogen atoms in the NH_2_ group and N1, C1, and C2 species. The definition of these angles is presented in Figure 2S in the ESI. The overlay of the allylamine molecules, differently colored for each adduct structure obtained at lower temperatures, is presented in Figure 6. All of the molecules are selected to have the vinyl group rotation in the range from 0° to 180°, which in some cases is associated with the inversion of the amine through the center of symmetry. The dihedral angle values for the amine molecule from each adduct are given in Table 3S in the ESI. To check how the geometry of the amine observed in the adducts corresponds to the isolated analogue *in vacuo*, potential energy scan for the optimized molecule, as well as amino and vinyl group rotations were performed. As the allylamine molecule is not chiral, the obtained potential energy surface (PES) was merged over the center of symmetry and is presented in Figure 6 in the form of a density plot. The comparison between the original and merged PES is presented in Figure 3S in the ESI. The global minimum of the isolated amine molecule occurs for torsion angles of about 126 and 124° for the vinyl and amine rotations, respectively. Among the amine molecules from the adducts, the closest geometry to this global minimum is observed for the structures containing EtOH, 1OctOH, 1NonOH, and 1DecOH. The other structures contain allylamine species located in the local minima of the PES. Interestingly, while for the structures with the T4(2) and L6(6) motifs, the amine geometry is almost the same for the structures with the identical topology, in the case of the adducts with the three smallest alcohols, the points representing the amine geometry are all located in different minima. This can be associated with the amine geometry adjusting to the L4(4)8(8) layer thickness, which is dependent on the alcohol chain length. Hence, in the case of the adducts with the shortest alcohols, MeOH and EtOH, the amine tends to be more compressed and folds to the geometry with coplanar NH_2_ and vinyl groups. For the longer 1-PrOH, the amine needs to be longer and has the vinyl group rotated by ca. 120°. In all of the 1D structures, allylamine is folded again (vinyl group rotation yielding ca. 0°). Such a geometry allows the terminal =CH_2_ moieties to better fill the space along the zigzag-shaped ribbon core. In addition, this also results in each ribbon being more compact. Finally, the last three adducts contain unfolded amine molecules with the geometry closest to the global minimum. This is due to the looser packing of the molecules in layers (smaller ρ_2D_ than for L4(4)8(8) motif structures) giving the amine more freedom and the possibility to adopt energetically favored conformation. In this case, the interactions between −CH_2_– and =CH_2_ moieties of the neighboring molecules can also play a role.

### Melting Points of the Adducts of Allylamine with *n*-alcohols

In the series of compounds containing aliphatic chains, the well-known odd–even melting point alternation phenomenon occurs. This is visible for alkanes^70^ or alkylamines,^73^ where melting points of the even C atom-containing compounds are considerably higher than neighboring odd C atom molecules. This is due to the effectiveness of the packing of the centrosymmetric even C atom molecules in the crystal lattice. Such an alternation effect is also observed in adducts^74^ but sometimes in reversed order as well.^74,75^

**Figure 7:**
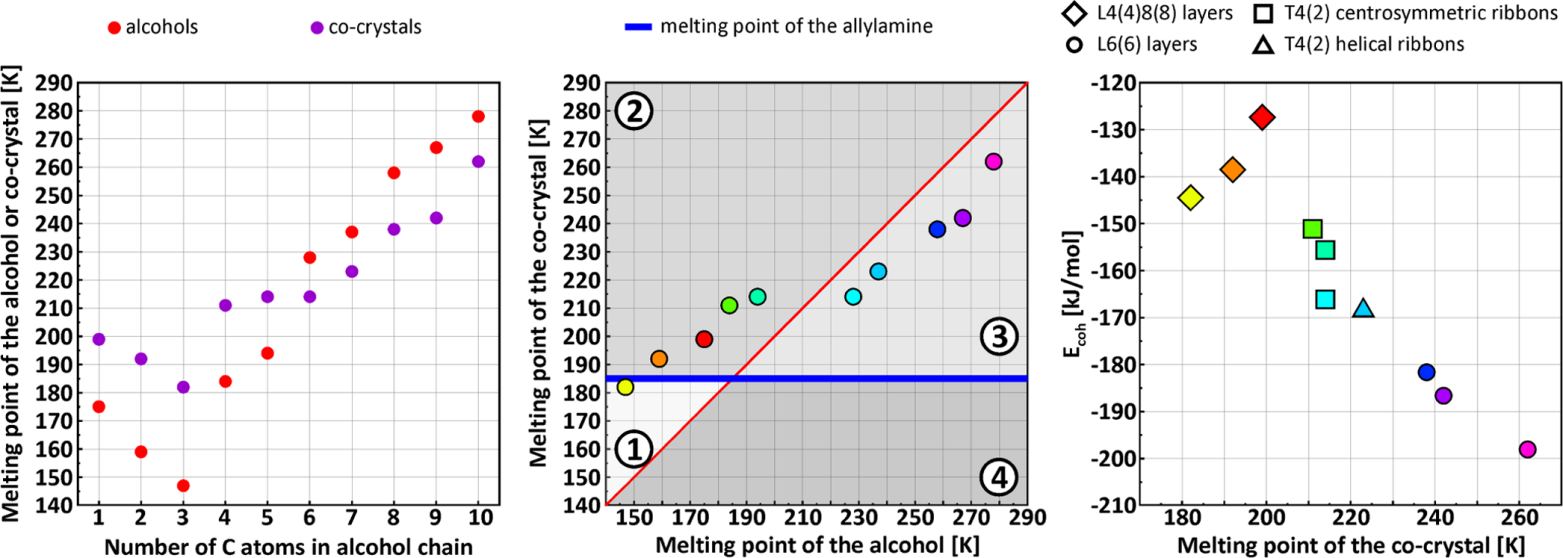
Comparison of the melting points of the allylamine adducts with alcohol chain length (left), melting points of the corresponding alcohols (middle), and cohesive energy of the system (right).

In the series of 10 adducts with allylamine presented here, there is no pronounced odd–even melting point alternation; moreover, crystals containing the three smallest alcohols tend to be thermally less stable with increasing carbon atom number in the alcohol moieties. This is clearly visible in the left diagram of Figure 7, which shows the melting point dependence on the C atom count, also including melting points of sole alcohols. However, some trends in the group of T4(2) topology adduct (reversed odd–even alternation for C4–C7) and L6(6) topology systems (classical odd–even alternation for C8–C10) can be observed. However, a surprising dependence is obtained when comparing the melting points of adducts and corresponding alcohols visualized in the middle diagram of Figure 7. Here, the horizontal blue line corresponds to the melting point of allylamine, whereas the red diagonal indicates the melting points of alcohols. The color scheme of the data points is the same as used in Figures 5 and 6. The diagram can be divided into four regions: ①—representing only one adduct with propanol, which is less stable than amine but more stable than the alcohol, ②—denoting systems more stable than the amine and alcohols, ③—phases more stable than the amine only, and ④—containing no data points, where adducts would be less stable than the pure ingredients. The obtained results slightly deviate from the observations based on the cocrystal melting point analysis performed by Perlovich.^46,76^ In the most recent paper by this author in the case of more than 2000 two-component molecular crystals, a group called “between (I)”, corresponding to regions ① + ③, is represented by 54.5%, group “higher (II)” corresponding to the region ② includes 14.2% of the cases, and group “low (III)” corresponding to the region ④ contains 31.1% structures. Among the amine–alcohol adducts presented here, the distribution is as follows: between (I), 60%; higher (II), 40%; and low(III), 0%. Such a discrepancy can be attributed to a very limited number of allylamine–alcohol adducts and the specificity of such dataset, where all of the systems contain the common ingredient. However, in both the cases, the majority belong to the between (I) group. Remarkably, the melting points of the adducts well correlate with the melting points of the alcohols. Interestingly, when the melting point of the amine and alcohol is comparable (or the difference is lower than ca. 30 K), the thermal stability of resulting adducts exceeds the melting points of both components. Once the difference is higher, such an adduct is less stable than either the amine or the alcohol. However, in the collection of molecular and multicomponent crystals, some prominent large melting point difference exceptions are known. One example is tetrahydrofuran clathrate hydrate,^77−80^ which melts at 277 K, while the pure components melt at 165 K (THF) and 273 K (ice). Such an amazing enhancement of the stability can be attributed here to encapsulation of THF by the 3D water framework, where entropy can play some role, and on the temperature increase, the energy will be consumed by exciting rotations and vibrations of THF moieties loosely bonded to the network but still sitting in the crystal lattice. Other good examples of enhanced stability of molecular multicomponent crystals are adducts of perfluorobenzene with benzene^81−83^ or mesitylene,^84−86^ which are stable at room temperature. In the case of the allylamine adducts with *n*-alcohols, however, the high melting point difference resulted in the crystallization of neat alcohols only when using 1:1 molar ratio mixtures. Successful crystallization of adducts containing 1-OctOH, 1-NonOH, and 1-DecOH was possible from liquids with doubled amounts of the amine, which decreases the thermal stability contributions of the crystalline alcohols. However, for the analyzed adducts, the melting points seem not to be dependent on the structural architecture. However, when the cohesive energy is plotted against the melting points in the right diagram of Figure 7, some clustering of data points representing different topology adducts can be observed.

## Conclusions

In conclusion, 10 new adducts containing allylamine and *n*-alcohols, in the methanol–decanol series, have been obtained using an IR laser-assisted, atmospheric pressure *in situ* crystallization method as all of these compounds are liquids under ambient conditions. These are the first adduct structures containing aliphatic *n*-alcohols and aliphatic amine (here allylamine) as co-formers. All of these structures are ordered, and the crystals indicate no phase transitions within their stability range down to a preset temperature of either 100 or 130 K, depending on the case. All of the phases contain the amine and alcohol in a 1:1 ratio; however, successful crystallization with the three biggest alcohols required an increased amount of the amine; otherwise, only the crystalline alcohols were formed. The three smallest alcohols crystallize with the allylamine to form centrosymmetric layers of molecules linked by hydrogen bonds. In these layers, four- and eight-membered rings involving NH_2_ and OH groups can be found giving the motif topology of the L4(4)8(8) type and synthons denoted as R_4_^2^(8) + R_8_^8^(16). Adducts with the next four alcohols contain molecules arranged in columns of the T4(2) type. In the cases of butanol, pentanol, and hexanol, the columns are centrosymmetric [R_4_^2^(8) and R_4_^4^(8) synthons], whereas, in the adducts with heptanol, they are chiral [R_4_^3^(8) ring motifs]. Nevertheless, all of these structures are centrosymmetric. Finally, the remaining three structures with the longest alcohols again have a 2D topology of the interacting molecules, but this time the arrangement is non-centrosymmetric. Here, the layers are of the L6(6) type with the NH_2_ and OH groups forming corrugated six-membered rings with the amine and alcohol chains located on opposite sides giving R_6_^5^(8) synthons. The structural investigations are supported by periodic calculations of layer (*E*_lbe_) and ribbon (*E*_rbe_) binding energies performed for isolated structural motifs from analyzed adducts, including reconstruction and application of these motifs to other *n*-alcohols, within the group, where feasible. The prediction of which structure will form based on the *E*_lbe_/*E*_rbe_ is complicated as the cohesive energy includes both the layer/ribbon binding energy and interlayer/inter-ribbon interaction energies. Each of these components changes differently with the elongation of the carbon chain. Moreover, the difficulty in calculating the value of the inter-ribbon interaction energy, which is dependent on the symmetry relationships between ribbons in the crystal lattice, makes it complicated to unequivocally determine for which chain length the 1D or 2D motif will be formed. The turning point of the motif change depends not only on the length of the alcohol carbon chain but also on the amine molecule, of course. Nevertheless, the centrosymmetric L4(4)8(8) layers with the adjacent amine and alcohol molecules are characteristic of comparable-sized alcohols with the amine species like methanol, ethanol, and propanol. With the longer alcohols, a columnar arrangement of the molecules appears. For a sufficiently long aliphatic chain in the *n*-alcohol, attractive interactions between CH_2_ moieties force the formation of layered architectures. As dispersive interactions begin to play an important role in the creation of non-centrosymmetric layers for longer *n*-alcohols, it may be anticipated that for branched-chain/cyclic alcohols or/and amines, 1D motifs will probably be preferred. Finally, in the analyzed series of 10 adducts, no melting point alternation was observed. Remarkably, the thermal stability of the adducts correlates with the melting points of the co-forming alcohols without evident relation to the adduct architecture. Furthermore, adducts with methanol, ethanol, butanol, and pentanol are thermally more stable than their crystalline co-formers.

## Supporting Information Available

Thermal ellipsoid plots for the allylamine n-alcohol adducts measured at the crystallization temperatures; hydrogen-bonding parameters and their calculate energies; *E*_rbe_/*E*_lbe_ values calculated for every possible structural motif; definition of the amine and vinyl group rotation angles in the allylamine molecule; amine and vinyl group rotation angles for the allylamine molecules; merged and unmerged density plot of the potential energy surface for the amine and vinyl groups 2π rotation; and melting points of the obtained adducts and melting points of the corresponding alcohols (PDF)

## References

[ref1] Pepinsky R. Crystal Engineering - New Concept in Crystallography. Phys. Rev. 1955, 100, 971.

[ref2] Desiraju G. R. Crystal Engineering: The Design of Organic Solids; Materials Science Monographs; Elsevier: Amsterdam; New York, 1989.

[ref3] Braga D. Crystal Engineering, Where from? Where To?. Chem. Commun. 2003, 2751 10.1039/b306269b. 14651093

[ref4] Cohen M. D.; Schmidt G. M. J. 383. Topochemistry. Part I. A Survey. J. Chem. Soc. Resumed 1964, 1996–2000. 10.1039/jr9640001996.

[ref5] Cohen M. D.; Schmidt G. M. J.; Sonntag F. I. 384. Topochemistry. Part II. The Photochemistry of Trans-Cinnamic Acids. J. Chem. Soc. Resumed 1964, 2000–2013. 10.1039/JR9640002000.

[ref6] Desiraju G. R. Crystal and Co-Crystal. CrystEngComm 2003, 5, 466–467. 10.1039/B313552G.

[ref7] Dunitz J. D. Crystal and Co-Crystal: A Second Opinion. CrystEngComm 2003, 5, 506 10.1039/B315687G.

[ref8] Aakeröy C. B.; Salmon D. J. Building Co-Crystals with Molecular Sense and Supramolecular Sensibility. CrystEngComm 2005, 7, 439–448. 10.1039/B505883J.

[ref9] Bond A. D. What Is a Co-Crystal?. CrystEngComm 2007, 9, 833–834. 10.1039/B708112J.

[ref10] Zaworotko M. J. Molecules to Crystals, Crystals to Molecules··· and Back Again?. Cryst. Growth Des. 2007, 7, 4–9. 10.1021/cg0680172.

[ref11] Aitipamula S.; Banerjee R.; Bansal A. K.; Biradha K.; Cheney M. L.; Choudhury A. R.; Desiraju G. R.; Dikundwar A. G.; Dubey R.; Duggirala N.; Ghogale P. P.; Ghosh S.; Goswami P. K.; Goud N. R.; Jetti R. R. K. R.; Karpinski P.; Kaushik P.; Kumar D.; Kumar V.; Moulton B.; Mukherjee A.; Mukherjee G.; Myerson A. S.; Puri V.; Ramanan A.; Rajamannar T.; Reddy C. M.; Rodriguez-Hornedo N.; Rogers R. D.; Row T. N. G.; Sanphui P.; Shan N.; Shete G.; Singh A.; Sun C. C.; Swift J. A.; Thaimattam R.; Thakur T. S.; Kumar Thaper R.; Thomas S. P.; Tothadi S.; Vangala V. R.; Variankaval N.; Vishweshwar P.; Weyna D. R.; Zaworotko M. J. Polymorphs, Salts, and Cocrystals: What’s in a Name?. Cryst. Growth Des. 2012, 12, 2147–2152. 10.1021/cg3002948.

[ref12] Yousef M. A. E.; Vangala V. R. Pharmaceutical Cocrystals: Molecules, Crystals, Formulations, Medicines. Cryst. Growth Des. 2019, 19, 7420–7438. 10.1021/acs.cgd.8b01898.

[ref13] Desiraju G. R. Crystal Engineering: A Holistic View. Angew. Chem., Int. Ed. 2007, 46, 8342–8356. 10.1002/anie.200700534. 17902079

[ref14] Ermer O.; Eling A. Molecular Recognition among Alcohols and Amines: Super-Tetrahedral Crystal Architectures of Linear Diphenol–Diamine Complexes and Aminophenols. J. Chem. Soc. Perkin Trans. 2 1994, 925–944. 10.1039/P29940000925.

[ref15] Allen F. H.; Hoy V. J.; Howard J. A. K.; Thalladi V. R.; Desiraju G. R.; Wilson C. C.; McIntyre G. J. Crystal Engineering and Correspondence between Molecular and Crystal Structures. Are 2- and 3-Aminophenols Anomalous?. J. Am. Chem. Soc. 1997, 119, 3477–3480. 10.1021/ja964254p.

[ref16] Vangala V. R.; Bhogala B. R.; Dey A.; Desiraju G. R.; Broder C. K.; Smith P. S.; Mondal R.; Howard J. A. K.; Wilson C. C. Correspondence between Molecular Functionality and Crystal Structures. Supramolecular Chemistry of a Family of Homologated Aminophenols. J. Am. Chem. Soc. 2003, 125, 14495–14509. 10.1021/ja037227p. 14624599

[ref17] Dey A.; Kirchner M. T.; Vangala V. R.; Desiraju G. R.; Mondal R.; Howard J. A. K. Crystal Structure Prediction of Aminols: Advantages of a Supramolecular Synthon Approach with Experimental Structures. J. Am. Chem. Soc. 2005, 127, 10545–10559. 10.1021/ja042738c. 16045342

[ref18] Ferguson G.; Glidewell C.; Gregson R. M.; Meehan P. R.; Patterson I. L. J. Formation of One-Dimensional Chains, Two-Dimensional Bilayers and a Three-Dimensional Diamondoid Architecture in Hydrogen-Bonded Adducts of 4,4′-Biphenol with 1,4-Diazabicyclo[2.2.2]Octane and 1,2-Diaminoethane. Acta Crystallogr., Sect. B: Struct. Sci., Cryst. Eng. Mater. 1998, 54, 151–161. 10.1107/S0108768197010148.

[ref19] Loehlin J. H.; Etter M. C.; Gendreau C.; Cervasio E. Hydrogen-Bond Patterns in Several 2:1 Amine-Phenol Cocrystals. Chem. Mater. 1994, 6, 1218–1221. 10.1021/cm00044a020.

[ref20] Shan N.; Bond A. D.; Jones W. Supramolecular Synthons in the Co-Crystal Structures of 2-Aminopyrimidine with Diols and Carboxylic Acids. Tetrahedron Lett. 2002, 43, 3101–3104. 10.1016/S0040-4039(02)00511-7.

[ref21] Hanessian S.; Gomtsyan A.; Simard M.; Roelens S. Molecular Recognition and Self-Assembly by “Weak” Hydrogen Bonding: Unprecedented Supramolecular Helicate Structures from Diamine/Diol Motifs. J. Am. Chem. Soc. 1994, 116, 4495–4496. 10.1021/ja00089a056.

[ref22] Hanessian S.; Saladino R.; Margarita R.; Simard M. Supramolecular Chirons Based on Enantiodifferentiating Self-Assembly between Amines and Alcohols (Supraminols). Chem. – Eur. J. 1999, 5, 2169–2183. 10.1002/(SICI)1521-3765(19990702)5:7<2169::AID-CHEM2169>3.0.CO;2-E.

[ref23] Hanessian S.; Simard M.; Roelens S. Molecular Recognition and Self-Assembly by Non-Amidic Hydrogen Bonding. An Exceptional Assembler of Neutral and Charged Supramolecular Structures. J. Am. Chem. Soc. 1995, 117, 7630–7645. 10.1021/ja00134a007.

[ref24] Scott J. L.; Hachiken S.; Tanaka K. Efficient Isomeric Enrichment in Cocrystals of Cyclohexanediamines and Low Molecular Weight Diols. Cryst. Growth Des. 2008, 8, 2447–2452. 10.1021/cg800032m.

[ref25] Mondal R.; Howard J. A. K.; Banerjee R.; Desiraju G. R. Crystallographic Studies of Supramolecular Synthons in Amine Solvates of *Trans*-1,5-Dichloro-9,10-Diethynyl-9,10-Dihydroanthracene-9,10-Diol. Cryst. Growth Des. 2006, 6, 2507–2516. 10.1021/cg060258m.

[ref26] Gdaniec M. On the Polymorphs of Pentafluorophenol and Its 2: 1 Co-Crystal with Pentafluoroaniline. CrystEngComm 2007, 9, 286–288. 10.1039/B700245A.

[ref27] Kirchner M. T.; Bläser D.; Boese R.; Desiraju G. R. Additive Induced Polymorphism. The Pentafluorophenol–Pentafluoroaniline System. CrystEngComm 2009, 11, 229–231. 10.1039/B810088H.

[ref28] Bellingen I. V.; Germain G.; Piret P.; Meerssche M. V. Structure Cristalline de Complexes Aniline–Phénol. I. Aniline–2,4,5-Trichlorophénol. Acta Crystallogr., Sect. B: Struct. Sci., Cryst. Eng. Mater. 1971, 27, 553–559. 10.1107/S0567740871002541.

[ref29] Vangala V. R.; Mondal R.; Broder C. K.; Howard J. A. K.; Desiraju G. R. Dianiline-Diphenol Molecular Complexes Based on Supraminol Recognition. Cryst. Growth Des. 2005, 5, 99–104. 10.1021/cg049967v.

[ref30] Loehlin J. H.; Franz K. J.; Gist L.; Moore R. H. Supramolecular Alcohol–Amine Crystals and Their Hydrogen-Bond Patterns. Acta Crystallogr., Sect. B: Struct. Sci., Cryst. Eng. Mater. 1998, 54, 695–704. 10.1107/S0108768198003231.

[ref31] Mootz D.; Brodalla D.; Wiebcke M. Structures of Monoethanolamine (MEAM), Diethanolamine (DEAM) and Triethanolamine (TEAM). Acta Crystallogr., Sect. C: Cryst. Struct. Commun. 1989, 45, 754–757. 10.1107/S0108270188013332.

[ref32] Choudhury A. R.; Yufit D. S.; Howard J. A. K. In situ Co-Crystallization of Cresols with Aniline and Fluoroanilines: Subtle Interplay of Strong and Weak Hydrogen Bonds. Z. Kristallogr. - Cryst. Mater. 2014, 229, 625–634. 10.1515/zkri-2014-1729.

[ref33] Gajda R.; Katrusiak A. Pressure-Freezing with Conformational Conversion of 3-Aminopropan-1-ol Molecules. Acta Crystallogr., Sect. B: Struct. Sci., Cryst. Eng. Mater. 2008, 64, 476–482. 10.1107/S0108768108012779. 18641449

[ref34] Abramov Y. A. On the Possibility of Kinetic Energy Density Evaluation from the Experimental Electron-Density Distribution. Acta Crystallogr., Sect. A: Found. Adv. 1997, 53, 264–272. 10.1107/S010876739601495X.

[ref35] Espinosa E.; Alkorta I.; Rozas I.; Elguero J.; Molins E. About the Evaluation of the Local Kinetic, Potential and Total Energy Densities in Closed-Shell Interactions. Chem. Phys. Lett. 2001, 336, 457–461. 10.1016/S0009-2614(01)00178-6.

[ref36] Apostolakis J.; Hofmann D. W. M.; Lengauer T. Derivation of a Scoring Function for Crystal Structure Prediction. Acta Crystallogr., Sect. A: Found. Adv. 2001, 57, 442–450. 10.1107/S0108767301004810. 11418755

[ref37] Gavezzotti A. Calculation of Intermolecular Interaction Energies by Direct Numerical Integration over Electron Densities. I. Electrostatic and Polarization Energies in Molecular Crystals. J. Phys. Chem. B 2002, 106, 4145–4154. 10.1021/jp0144202.

[ref38] Dunitz J. D.; Gavezzotti A. Supramolecular Synthons: Validation and Ranking of Intermolecular Interaction Energies. Cryst. Growth Des. 2012, 12, 5873–5877. 10.1021/cg301293r.

[ref39] Brezgunova M. E.; Aubert E.; Dahaoui S.; Fertey P.; Lebègue S.; Jelsch C.; Ángyán J. G.; Espinosa E. Charge Density Analysis and Topological Properties of Hal_3_-Synthons and Their Comparison with Competing Hydrogen Bonds. Cryst. Growth Des. 2012, 12, 5373–5386. 10.1021/cg300978x.

[ref40] Gavezzotti A. The “Sceptical Chymist”: Intermolecular Doubts and Paradoxes. CrystEngComm 2013, 15, 4027 10.1039/c3ce00051f.

[ref41] Shishkin O. V.; Zubatyuk R. I.; Shishkina S. V.; Dyakonenko V. V.; Medviediev V. V. Role of Supramolecular Synthons in the Formation of the Supramolecular Architecture of Molecular Crystals Revisited from an Energetic Viewpoint. Phys. Chem. Chem. Phys. 2014, 16, 6773–6786. 10.1039/C3CP55390F. 24595277

[ref42] Stepanovs D.; Jure M.; Kuleshova L. N.; Hofmann D. W. M.; Mishnev A. Cocrystals of Pentoxifylline: In Silico and Experimental Screening. Cryst. Growth Des. 2015, 15, 3652–3660. 10.1021/acs.cgd.5b00185.

[ref43] Zolotarev P. N.; Moret M.; Rizzato S.; Proserpio D. M. Searching New Crystalline Substrates for OMBE: Topological and Energetic Aspects of Cleavable Organic Crystals. Cryst. Growth Des. 2016, 16, 1572–1582. 10.1021/acs.cgd.5b01695.

[ref44] Mackenzie C. F.; Spackman P. R.; Jayatilaka D.; Spackman M. A. CrystalExplorer Model Energies and Energy Frameworks: Extension to Metal Coordination Compounds, Organic Salts, Solvates and Open-Shell Systems. IUCrJ 2017, 4, 575–587. 10.1107/S205225251700848X. PMC5600021 28932404

[ref45] Dziuk B.; Gianopoulos C. G.; Ejsmont K.; Zarychta B. Self-Assembly Mechanism Based on Charge Density Topological Interaction Energies. Struct. Chem. 2018, 29, 703–713. 10.1007/s11224-017-1060-6.

[ref46] Perlovich G. Melting Points of One- and Two-Component Molecular Crystals as Effective Characteristics for Rational Design of Pharmaceutical Systems. Acta Crystallogr. Sect. B Struct. Sci. Cryst. Eng. Mater. 2020, 76, 696–706. 10.1107/S2052520620007362. 32831288

[ref47] Boese R. Special Issue on *In Situ* Crystallization. Z. Kristallogr. - Cryst. Mater. 2014, 229, 595–601. 10.1515/zkri-2014-5003.

[ref48] APEX3 Software Package V2019; Bruker AXS Inc.: Madison, WI, 2019.

[ref49] Bruker SAINT, v8.40A: Part of the APEX3 Software Package V2019; Bruker AXS Inc.: Madison, WI, 2019.

[ref50] Bruker SADABS V2016/2: Part of the APEX3 Software Package V2019; Bruker AXS Inc.: Madison, WI, 2019.

[ref51] Sheldrick G. M. *SHELXT* – Integrated Space-Group and Crystal-Structure Determination. Acta Crystallogr., Sect. A: Found. Adv. 2015, 71, 3–8. 10.1107/S2053273314026370. 25537383 PMC4283466

[ref52] Sheldrick G. M. Crystal Structure Refinement with SHELXL. Acta Crystallogr., Sect. C: Cryst. Struct. Commun. 2015, 71, 3–8. 10.1107/S2053229614024218. PMC4294323 25567568

[ref53] International Tables for Crystallography: Mathematical, Physical and Chemical Tables, 1st ed.; Prince E.; Fuess H.; Hahn Th.; Wondratschek H.; Müller U.; Shmueli U.; Prince E.; Authier A.; Kopský V.; Litvin D. B.; Rossmann M. G.; Arnold E.; Hall S.; McMahon B., Series Eds.; International Tables for Crystallography; International Union of Crystallography: Chester, England, 2006; Vol. C 10.1107/97809553602060000103.

[ref54] Putz H.; Brandenburg K. Diamond - Crystal and Molecular Structure Visualization, Crystal Impact; https://www.crystalimpact.de/diamond.

[ref55] Macrae C. F.; Bruno I. J.; Chisholm J. A.; Edgington P. R.; McCabe P.; Pidcock E.; Rodriguez-Monge L.; Taylor R.; Streek J.; van de Wood P. A. *Mercury CSD 2.0* – New Features for the Visualization and Investigation of Crystal Structures. J. Appl. Crystallogr. 2008, 41, 466–470. 10.1107/S0021889807067908.

[ref56] Hohenberg P.; Kohn W. Inhomogeneous Electron Gas. Phys. Rev. 1964, 136, B864–B871. 10.1103/PhysRev.136.B864.

[ref57] Parr R. G.; Weitao Y. Density-Functional Theory of Atoms and Molecules; International Series of Monographs on Chemistry; Oxford University Press: New York, 1995. 10.1093/oso/9780195092769.001.0001.

[ref58] Dovesi R.; Erba A.; Orlando R.; Zicovich-Wilson C. M.; Civalleri B.; Maschio L.; Rérat M.; Casassa S.; Baima J.; Salustro S.; Kirtman B. Quantum-Mechanical Condensed Matter Simulations with CRYSTAL. WIREs Comput. Mol. Sci. 2018, 8, e1360 10.1002/wcms.1360.

[ref59] Dovesi R.; Saunders V. R.; Roetti C.; Orlando R.; Zicovich-Wilson C. M.; Pascale F.; Civalleri B.; Doll K.; Harrison N. M.; Bush I. J.; D’Arco P.; lunell M.; Causà M.; Noël Y.; Maschio L.; Erba A.; Rerat M.; Casassa S. CRYSTAL17 User’s Manual 2017.

[ref60] Grimme S.; Antony J.; Ehrlich S.; Krieg H. A Consistent and Accurate Ab Initio Parametrization of Density Functional Dispersion Correction (DFT-D) for the 94 Elements H-Pu. J. Chem. Phys. 2010, 132, 154104 10.1063/1.3382344. 20423165

[ref61] Brandenburg J. G.; Grimme S. Dispersion Corrected Hartree–Fock and Density Functional Theory for Organic Crystal Structure Prediction. In Prediction and Calculation of Crystal Structures: Methods and Applications. Topics in Current Chemistry, Atahan-Evrenk S.; Aspuru-Guzik A., Eds.; Springer International Publishing: Cham, 2014; pp 1–23 10.1007/128_2013_488. 24220994

[ref62] Boys S. F.; Bernardi F. The Calculation of Small Molecular Interactions by the Differences of Separate Total Energies. Some Procedures with Reduced Errors. Mol. Phys. 1970, 19, 553–566. 10.1080/00268977000101561.

[ref63] Socha P.; Prus B.; Dobrzycki Ł.; Boese R.; Cyrański M. K. Intermolecular Interactions in Hydrates of 4-Methylpiperidine and 4-Chloropiperidine – a Structural and Computational Study. CrystEngComm 2021, 23, 1251–1262. 10.1039/D0CE01585G.

[ref64] Frisch M. J.; Trucks G. W.; Schlegel H. B.; Scuseria G. E.; Robb M. A.; Cheeseman J. R.; Scalmani G.; Barone V.; Petersson G. A.; Nakatsuji H.; Li X.; Caricato M.; Marenich A. V.; Bloino J.; Janesko B. G.; Gomperts R.; Mennucci B.; Hratchian H. P.; Ortiz J. V.; Izmaylov A. F.; Sonnenberg J. L.; Williams-Young D.; Ding F.; Lipparini F.; Egidi F.; Goings J.; Peng B.; Petrone A.; Henderson T.; Ranasinghe D.; Zakrzewski V. G.; Gao J.; Rega N.; Zheng G.; Liang W.; Hada M.; Ehara M.; Toyota K.; Fukuda R.; Hasegawa J.; Ishida M.; Nakajima T.; Honda Y.; Kitao O.; Nakai H.; Vreven T.; Throssell K.; Montgomery J. A. Jr.; Peralta J. E.; Ogliaro F.; Bearpark M. J.; Heyd J. J.; Brothers E. N.; Kudin K. N.; Staroverov V. N.; Keith T. A.; Kobayashi R.; Normand J.; Raghavachari K.; Rendell A. P.; Burant J. C.; Iyengar S. S.; Tomasi J.; Cossi M.; Millam J. M.; Klene M.; Adamo C.; Cammi R.; Ochterski J. W.; Martin R. L.; Morokuma K.; Farkas O.; Foresman J. B.; Fox D. J.. Gaussian 16, Revision C.01; Gaussian Inc.: Wallingford, CT, 2016.

[ref65] Infantes L.; Motherwell S. Water Clusters in Organic Molecular Crystals. CrystEngComm 2002, 4, 454–461. 10.1039/B204934A.

[ref66] Saenger W.; Lindner K. OH Clusters with Homodromic Circular Arrangement of Hydrogen Bonds. Angew. Chem., Int. Ed. 1980, 19, 398–399. 10.1002/anie.198003981.

[ref67] Etter M. C. Encoding and Decoding Hydrogen-Bond Patterns of Organic Compounds. Acc. Chem. Res. 1990, 23, 120–126. 10.1021/ar00172a005.

[ref68] Dobrzycki L.; Woźniak K. Structures of Hybrid Inorganic–Organic Salts with Benzidine Dication Derivatives. CrystEngComm 2008, 10, 525–533. 10.1039/B714587J.

[ref69] Dobrzycki L.; Woźniak K. 1D vs 2D Crystal Architecture of Hybrid Inorganic–Organic Structures with Benzidine Dication. J. Mol. Struct. 2009, 921, 18–33. 10.1016/j.molstruc.2008.12.027.

[ref70] Boese R.; Weiss H.-C.; Bläser D. The Melting Point Alternation in the Short-Chain *n*-Alkanes: Single-Crystal X-Ray Analyses of Propane at 30 K and of *n*-Butane to *n*-Nonane at 90 K. Angew. Chem., Int. Ed. 1999, 38, 988–992. 10.1002/(SICI)1521-3773(19990401)38:7<988::AID-ANIE988>3.0.CO;2-0. 29711877

[ref71] Bond A. D.; Davies J. E. *N*-Decane. Acta Crystallogr., Sect. E Struct. Rep. Online 2002, 58, o196–o197. 10.1107/S1600536802001332.

[ref72] Shallard-Brown H. A.; Watkin D. J.; Cowley A. R. *N*-Octanol. Acta Crystallogr., Sect. E Struct. Rep. Online 2005, 61, o213–o214. 10.1107/S1600536804032775.

[ref73] Maloney A. G. P.; Wood P. A.; Parsons S. Competition between Hydrogen Bonding and Dispersion Interactions in the Crystal Structures of the Primary Amines. CrystEngComm 2014, 16, 3867–3882. 10.1039/C3CE42639D.

[ref74] Braga D.; Dichiarante E.; Palladino G.; Grepioni F.; Chierotti M. R.; Gobetto R.; Pellegrino L. Remarkable Reversal of Melting Point Alternation by Co-Crystallization. CrystEngComm 2010, 12, 3534–3536. 10.1039/C0CE00253D.

[ref75] Bond A. D. Inversion of the Melting Point Alternation in *n*-Alkyl Carboxylic Acids by Co-Crystallization with Pyrazine. CrystEngComm 2006, 8, 333–337. 10.1039/B517513E.

[ref76] Perlovich G. L. Two-Component Molecular Crystals: What Is the Difference between Drug–Drug, Drug–GRAS, and CF–CF Databases? Evaluation of Melting Points and Ideal Solubility of Unknown Co-Crystals. Cryst. Growth Des. 2021, 21, 5058–5071. 10.1021/acs.cgd.1c00477.

[ref77] Stackelberg M.; Meuthen B. Feste Gashydrate. VII. Hydrate Wasserlöslicher Äther. Z. Für Elektrochem. Berichte Bunsenges. Für Phys. Chem. 1958, 6, 130–131.

[ref78] Davidson D. W.; Davies M. M.; Williams K. Dielectric Absorption and Molecular Motion in Gas Hydrates. J. Chem. Phys. 1964, 40, 3449–3450. 10.1063/1.1725032.

[ref79] Sargent D. F.; Calvert L. D. Crystallographic Data for Some New Type II Clathrate Hydrates^1^. J. Phys. Chem. A 1966, 70, 2689–2691. 10.1021/j100880a503.

[ref80] Dobrzycki L.; Taraszewska P.; Boese R.; Cyrański M. K. Pyrrolidine and Its Hydrates in the Solid State. Cryst. Growth Des. 2015, 15, 4804–4812. 10.1021/acs.cgd.5b00527.

[ref81] Overell J. S. W.; Pawley G. S. An X-Ray Single-Crystal Study of the Molecular System C_6_F_6_.C_6_D_6_. Acta Crystallogr., Sect. B: Struct. Sci., Cryst. Eng. Mater. 1982, 38, 1966–1972. 10.1107/S0567740882007687.

[ref82] Williams J. H.; Cockcroft J. K.; Fitch A. N. Structure of the Lowest Temperature Phase of the Solid Benzene–Hexafluorobenzene Adduct. Angew. Chem., Int. Ed. 1992, 31, 1655–1657. 10.1002/anie.199216551.

[ref83] Cockcroft J. K.; Rosu-Finsen A.; Fitch A. N.; Williams J. H. The Temperature Dependence of C–H···F–C Interactions in Benzene: Hexafluorobenzene. CrystEngComm 2018, 20, 6677–6682. 10.1039/C8CE01187G.

[ref84] Dahl T.; Gropen O.; Wilhelmi K.-A.; Lindberg A. A.; Lagerlund I.; Ehrenberg L. Crystal Structure of the 1:1 Complex between Mesitylene and Hexafluorobenzene. Acta Chem. Scand. 1971, 25, 1031–1039. 10.3891/acta.chem.scand.25-1031.

[ref85] Cockcroft J. K.; Ghosh R. E.; Shephard J. J.; Singh A.; Williams J. H. Investigation of the Phase Behaviour of the 1:1 Adduct of Mesitylene and Hexafluorobenzene. CrystEngComm 2017, 19, 1019–1023. 10.1039/C6CE02581A.

[ref86] Timmer B. J. J.; Mooibroek T. J. Intermolecular π–π Stacking Interactions Made Visible. J. Chem. Educ. 2021, 98, 540–545. 10.1021/acs.jchemed.0c01252. 33583952 PMC7876799

