# Supplementary material for: Hydrogen-Bonding Motifs in Adducts of Allylamine with the 10 Simplest n-Alcohols: Single-Crystal X-ray Diffraction Studies and Computational Analysis
Source: Cryst Growth Des. 2022 Oct 11;22(11):6405–17. doi: 10.1021/acs.cgd.2c00316 (PMC9635617; doi:10.1021/acs.cgd.2c00316)
Supplement: Supplementary file 1 — cg2c00316_si_001.pdf [file cg2c00316_si_001.pdf]

Hydrogen bond motifs in adducts of allylamine with the ten simplest *n*−alcohols: single crystal X−ray diffraction studies and computational analysis

Bernadeta Prus[1,2], Michał K. Cyrański[1], Roland Boese[1], Janusz Zachara[2], Łukasz Dobrzycki[1]

[1]Laboratory of Advanced Crystal Engineering, Faculty of Chemistry, University of Warsaw, Żwirki i Wigury 101, 02-089 Warsaw, Poland

[2]Faculty of Chemistry, Warsaw University of Technology, ul. Noakowskiego 3, 00-664 Warsaw, Poland

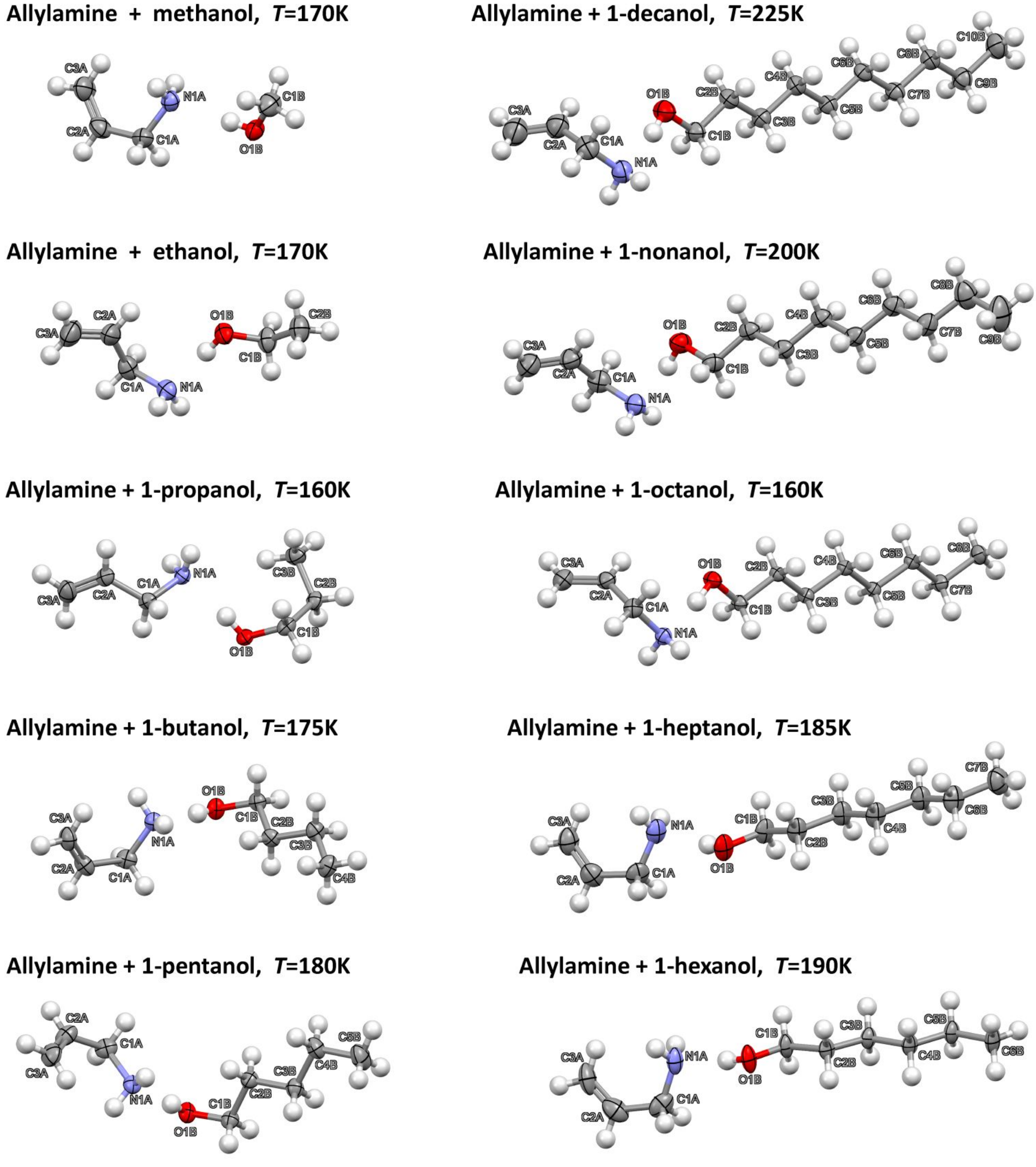

Figure 1S. Displacement ellipsoid plots (drawn at 50% probability level) and numbering scheme for the allylamine *n*-alcohol adducts measured at at the crystallization temperatures.

Table 1S. Hydrogen bond parameters and their calculate energies (E_{pair}) in studied allylamine adducts with *n*-alcohols.

Adduct	Bond	D-H (Å)	A-H (Å)	D-A (Å)	Angle (deg)	E_{pair} (kJ/mol)
**MeOH**	O1B-H1OB···N1A	0.837(17)	1.932(17)	2.7683(11)	176.9(14)	-28.57
	N1A-H1NA···O1B	0.873(15)	2.241(15)	3.1073(11)	171.5(12)	-14.39
	N1A-H2NA···O1B	0.874(14)	2.307(14)	3.1343(12)	158.0(11)	-15.02
**EtOH**	O1B-H1OB···N1A	0.93(2)	1.82(2)	2.7517(17)	176(2)	-28.43
	N1A-H1NA···O1B	0.849(19)	2.29(2)	3.1358(18)	175.3(16)	-17.82
	N1A-H2NA···O1B	0.88(2)	2.22(2)	3.0957(17)	174.1(18)	-12.78
**1-PrOH**	O1B-H1OB···N1A	0.866(18)	1.904(18)	2.7666(12)	174.5(15)	-27.24
	N1A-H1NA···O1B	0.879(15)	2.274(16)	3.1405(12)	168.9(12)	-15.54
	N1A-H2NA···O1B	0.887(14)	2.377(15)	3.1921(13)	153.0(12)	-16.42
**1-BuOH**	O1B-H1OB···N1A	0.85(2)	1.94(2)	2.7952(13)	176.0(16)	-34.61
	N1A-H1NA···O1B	0.905(18)	2.293(18)	3.1959(13)	176.3(13)	-14.63
	N1A-H2NA···O1B	0.879(18)	2.293(18)	3.1608(14)	169.6(14)	-17.12
**1-PentOH**	O1B-H1OB···N1A	0.868(19)	1.916(19)	2.7829(12)	176.5(16)	-34.24
	N1A-H1NA···O1B	0.907(17)	2.244(17)	3.1506(12)	178.6(13)	16.83
	N1A-H2NA···O1B	0.915(16)	2.303(16)	3.2052(13)	168.7(12)	-14.24
**1-HexOH**	O1B-H1OB···N1A	0.87(3)	1.93(3)	2.7908(15)	174(2)	-35.06
	N1A-H1NA···O1B	0.87(2)	2.32(2)	3.1866(17)	171.4(18)	-16.67
	N1A-H2NA···O1B	0.88(2)	2.31(2)	3.1655(17)	164.0(17)	-18.13
**1-HeptOH**	O1B-H1OB···N1A	0.79(4)	2.02(4)	2.794(3)	170(3)	-35.26
	N1A-H1NA···O1B	0.88(4)	2.33(4)	3.199(3)	167(3)	-15.30
	N1A-H2NA···O1B	0.88(3)	2.31(3)	3.187(3)	174(2)	-14.54
**1-OctOH**	O1B-H1OB···N1A	0.92(3)	1.85(3)	2.7668(18)	173(2)	-37.95
	N1A-H1NA···O1B	0.89(2)	2.42(2)	3.261(2)	158.3(18)	-13.60
	N1A-H2NA···O1B	0.90(2)	2.17(2)	3.0625(19)	167.3(19)	-14.04
**1-NonOH**	O1B-H1OB···N1A	0.98(3)	1.77(3)	2.7416(15)	171(2)	-37.98
	N1A-H1NA···O1B	0.897(19)	2.16(2)	3.0335(15)	166.1(16)	-13.67
	N1A-H2NA···O1B	0.87(2)	2.39(2)	3.2168(16)	158.0(15)	-13.34
**1-DecOH**	O1B-H1OB···N1A	0.91(4)	1.87(4)	2.768(3)	173(3)	-37.95
	N1A-H1NA···O1B#2	0.92(3)	2.39(3)	3.257(3)	159(2)	-13.63
	N1A-H2NA···O1B#1	0.93(3)	2.14(3)	3.060(3)	166(3)	-14.05

Table 2S. E_{rbe}/E_{lbe} values calculated for every possible structural motif - optimized based on the observed structures (green), created (red).

Adduct	MeOH	EtOH	1-PrOH	1-BuOH	1-PentOH	1-HexOH	1-HeptOH	1-OctOH	1-NonOH	1-DecOH
E_{lbe} $R_4^2(8) + R_8^8(16)$	-119.48	-130.33	-129.24	-134.35	-	-	-	-	-	-
E_{rbe} $R_4^4(8) + R_4^2(8)$	-106.08	-103.30	-105.99	-110.36	-113.71	-109.96	-112.26	-113.78	-116.02	-117.75
E_{rbe} $R_4^3(8)$	-98.96	-107.13	-110.77	-115.84	-114.06	-113.77	-115.65	-117.57	-119.44	-121.26
E_{lbe} $R_6^5(12)$	-113.28	-121.43	-130.05	-138.34	-146.04	-153.96	-158.85	-168.31	-175.70	-184.15

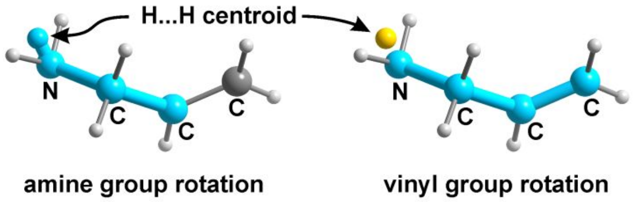

Figure 2S. Definition of the amine and vinyl group rotation angles in the allylamine molecule.

Table 3S. Amine and vinyl group rotation angles for the allylamine molecules in adducts with the given alcohol.

Adduct	Amine group rotation /°	Vinyl group rotation /°
**MeOH**	-10.64	1.99058
**EtOH**	-130.72	122.10111
**1-PrOH**	11.11	126.22041
**1-BuOH**	7.74	3.98883
**1-PentOH**	6.94	3.71197
**1-HexOH**	-5.98	3.04693
**1-HeptOH**	-11.32	4.81670
**1-OctOH**	-129.13	122.94820
**1-NonOH**	-128.25	122.73494
**1-DecOH**	-128.81	123.04579

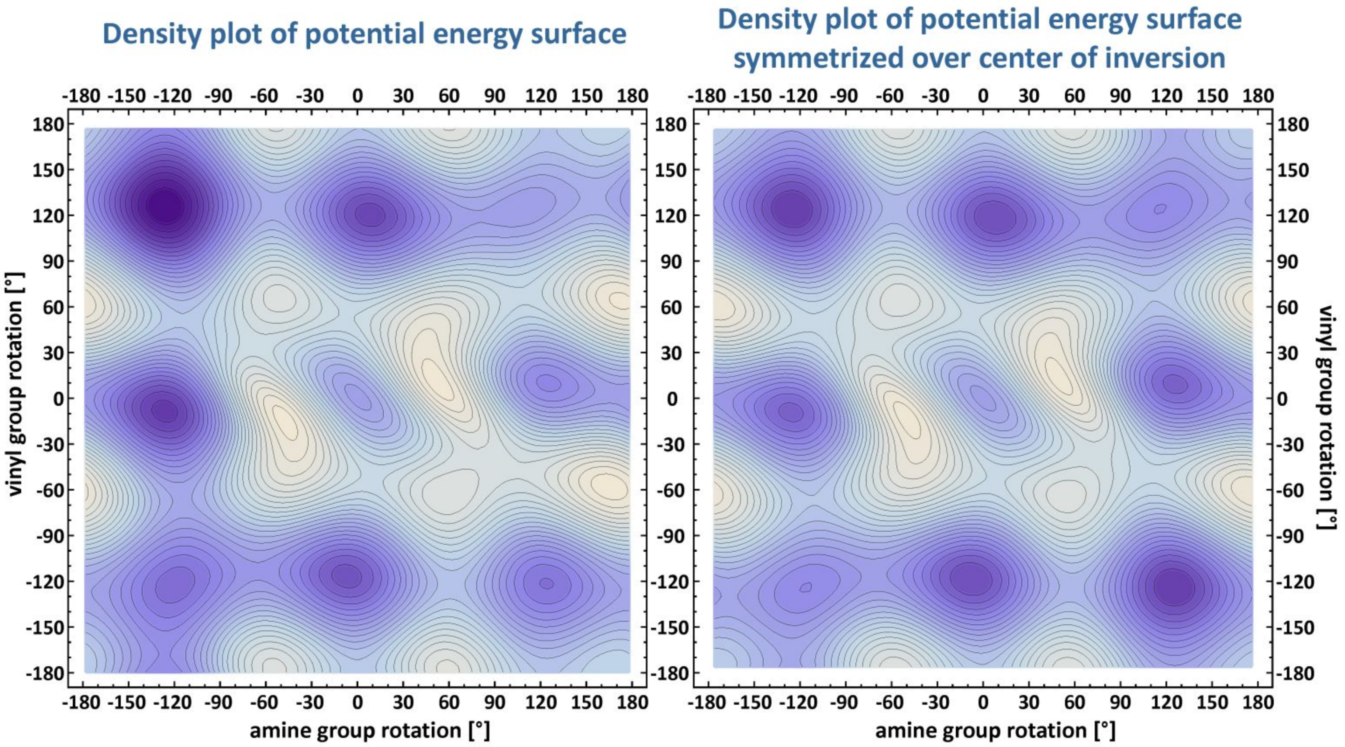

Figure 3S. Density plot of potential energy surface for amine and vinyl groups 2π rotation – unmerged (full 2π range rotation over two angles) – left, and merged over center of inversion – right. Both pictures at the same color scale, the darker color the deeper minima, contours every 1.0502 kJ/mol.

Table 4S. Melting points of the obtained adducts and melting points of the corresponding alcohols.

Adduct	Melting point of the adduct	Melting point of the alcohol
**MeOH**	199K	175K
**EtOH**	192K	159K
**1-PrOH**	182K	147K
**1-BuOH**	211K	184K
**1-PentOH**	214K	194K
**1-HexOH**	214K	228K
**1-HeptOH**	223K	237K
**1-OctOH**	238K	258K
**1-NonOH**	242K	267K
**1-DecOH**	262K	278K
